# Physiological Plasticity and Growth Dynamics as Predictive Parameters for Screening Salinity Stress Gradient Responses in Four *Triticum aestivum* L. Varieties: Boema, Glosa, Granny and Taisa

**DOI:** 10.3390/plants15060867

**Published:** 2026-03-11

**Authors:** Mădălina Trușcă, Valentina Ancuța Stoian, Ștefania Gâdea, Anamaria Vâtcă, Vlad Stoian, Sorin Daniel Vâtcă

**Affiliations:** 1 Department of Plant Physiology, Faculty of Agriculture, University of Agricultural Sciences and Veterinary Medicine Cluj-Napoca, Calea Mănăştur 3–5, 400372 Cluj-Napoca, Romania; ioana-madalina.trusca@student.usamvcluj.ro (M.T.); stefania.gadea@usamvcluj.ro (Ș.G.); sorin.vatca@usamvcluj.ro (S.D.V.); 2 Department of Management and Economics, Faculty of Animal Science and Biotechnologies, University of Agricultural Sciences and Veterinary Medicine Cluj-Napoca, Calea Mănăştur 3–5, 400372 Cluj-Napoca, Romania; anamaria.vatca@usamvcluj.ro; 3 Department of Microbiology, Faculty of Agriculture, University of Agricultural Sciences and Veterinary Medicine Cluj-Napoca, Calea Mănăştur 3–5, 400372 Cluj-Napoca, Romania; vlad.stoian@usamvcluj.ro

**Keywords:** stomatal features, chlorophyll content, morphological parameters, Taisa, Boema, Granny, Glosa, salinity effect

## Abstract

Soil salinity in wheat represents a severe threat to global productivity, requiring a deep understanding of physiological adaptation mechanisms to ensure food security in the context of continuous agricultural land degradation. The study aim was to assess the impact of a salinity gradient (0–75 mM NaCl) on the dynamics of stomatal opening and chlorophyll content of the varieties Glosa, Taisa, Boema and Granny. The methodology integrated four joint classes, of which two were from detailed physiological parameters, stomatal features and chlorophyll content, and two morphological characteristics, growth visual indices and biomass allocation. All data was corroborated into an original hierarchical synthesis model presented in a multi-layered sunburst plot. The most relevant results indicate that the concentration of 45 mM NaCl represents the osmotic adjustment threshold, where the active accumulation of ions decreases the internal osmotic potential, facilitating an influx of water that maximizes guard cell turgor and, implicitly, stomatal width. Maximal physiological parameters and biomass ranked the variety Granny first, followed by Taisa. Despite stomatal increases, Boema ranked third and Glosa showed overall decreased development and the lowest plant biomass. These findings validate the use of interconnected effects analysis as a screening tool for identifying the salinity responses of wheat varieties.

## 1. Introduction

Ongoing transformations within agricultural ecosystems [1] supported by various new cropping technologies have led to production upscaling [2]. Currently, there are modern revolutionary methods that facilitate farmers’ efforts and improve the results of cropping [3]. However, it should be emphasized that, going beyond the practicality of plant cultivation, factual data utilization, potential problem mitigation and adaptation to changing environmental conditions are essential for agriculture [4].

The negative interaction between climate change and soil salinity poses a severe threat to crop production, affecting both crop yield and harvest quality [5]. Efficient resource management is essential to mitigate these effects and ensure global food security [6]. A fundamental component of such management is the physiological assessment of seed collection, which allows for the identification of specific varieties possessing superior adaptive characteristics and resilience to environmental stressors [7,8].

As the world’s population continues to grow, the challenge of ensuring global food security remains a critical uncertainty [9]. The crops that underpin global food supply are rice, maize and wheat [10,11]. Given their accessibility and central role in global nutrition, by providing carbohydrates, proteins and fibers [12], maintaining the productivity of these cereals is a vital agricultural priority.

However, climate change and all its consequences limit the cropping area of wheat, maize [13] and rice [14]. Therefore, there is a need to assess the stress tolerance and sensitivity of these globally important crops. Even if rice is also grown in regions with saline soils, the association of salinity with high temperatures caused by climate change has an adverse impact on crop growth and development [15]. Maize is sensitive to heat stress, which causes a significant decrease in yield. Similarly, in a saline environment, maize crop yields are affected by a reduction in plant height and thus the potential biomass [16].

Wheat is moderately sensitive to salinity [17]. The onset of osmotic stress of wheat is associated with toxicity [18] and altered physiological processes [19], which is reflected in decreased yield. Salinity produces interconnected effects [20], influencing chlorophyll pigments synthesis and photosynthetic capacity [21], leading to a growth reduction compromising biomass allocation [17]. This decline is reflected in smaller stems, reduced leaf area, and short and thin spikes due to the stress effect upon cell division and expansion [22]. Under saline conditions, photosynthesis is not only reduced by defective chlorophyll synthesis but also by the reduced gas exchange efficiency through stomata [23]. In the presence of NaCl, stomata are often closed to prevent water loss [24].

With over 30% of the world’s population depending on wheat as their main food source [17] and continued expansion of soil salinization [25], the study of different wheat germplasm becomes essential. Testing wheat varieties capable of maintaining productivity under salt stress is essential. Each variety, through the genetic background inherited, has its own threshold limits. Despite recent advances in wheat breeding, the assessment of existing varieties represents a cost-effective strategy in order to maintain genetic diversity for future agricultural challenges [26].

To address the need for identifying wheat sensitivity or tolerance to salinity stress, this study aimed to assess the morpho-physiological responses of four varieties to a salt solution gradient. The study followed a multi-objective framework: (i) to quantify the relative chlorophyll content during the most important phenophases of the entire growth season; (ii) to analyze stomatal imprints features in three different leaf sampling areas; (iii) to correlate the biomass and morphological characteristics under the impact of different doses of salinity; (iv) to integrate all data into a hierarchical synthesis model to define overall variety-specific response to different salinity stress doses.

## 2. Results

### 2.1. Wheat Stomata Characteristics

#### 2.1.1. The Effect of Wheat Variety × Salinity on Stomatal Density

**Figure 1:**
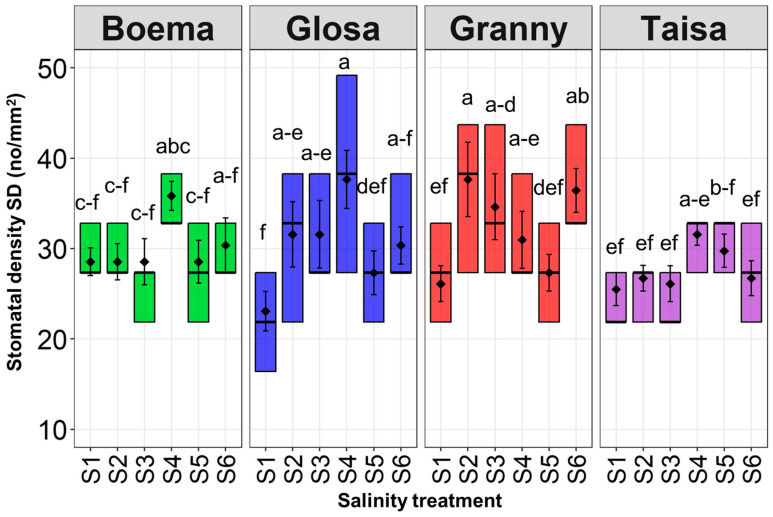
Stomatal density (SD) for each wheat variety tested under all concentrations (S1—control, S2—15 mM NaCl, S3—30 mM NaCl, S4—45 mM NaCl, S5—60 mM NaCl, and S6—75 mM NaCl of applied salt stress). Distinct lowercase letters denoting statistical significance at *p* < 0.05 based on LSD test.

**Figure A1:**
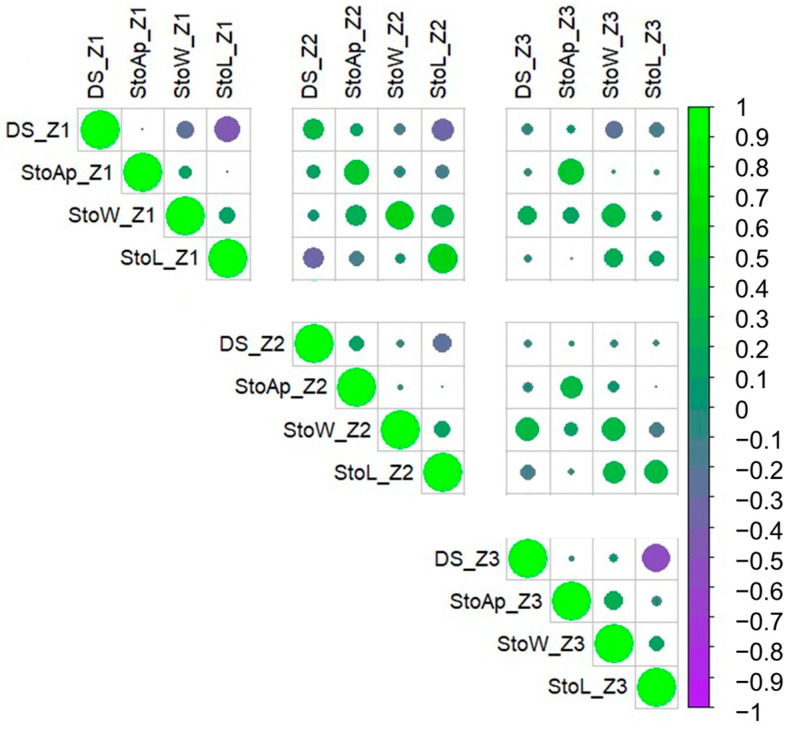
Correlogram between stomatal density (SD), stomata length (StoL), width (StoW), aperture (StoAP) and sampling area (A1—base, A2—middle, and A3—tip) within all varieties and all salinity doses.

The wheat stomatal density (SD) values varied greatly, depending on both the wheat variety tested and the NaCl concentration (Figure 1 and Figure A1).

Maximum values of 38 for SD were recorded for the Gr variety at the lowest saline concentration (S2) and for Gl at S4. These values were 1.6 times higher for Gl and 1.4 times higher for Gr compared to the corresponding control.

All four wheat varieties tested showed a trend of high stomatal density values in the range between 30 and 38 under S4. This was followed by a decrease, with around 18% of SD values at the S5 concentration for all varieties. This reduction of 10 units was significant only for Gl wheat. The trend was further followed by another increase in SD values, with 6% for Bo, with 11% for Gl, and with 33% for Gr at the highest saline dose (S6) compared to S5. Ta registered a decrease of 10% in SD with the increasing salinity dose to S6. For all the wheat varieties, the SD values increased with increasing salinity doses. Within the recorded treatment values, the Bo and Ta varieties showed a pattern of reduced differences between values recorded at salinity in the range of S1–S5, while at S6 the data collected showed a larger difference. The Gr and Gl varieties showed an opposite situation, with larger differences for values recorded within the same variety.

#### 2.1.2. The Effect of Wheat Variety × Salinity on Stomatal Length

Following the two-way ANOVA analysis, the variety factor determined significant statistical differences in stomatal length (StoL). The longest StoLs were recorded for the Ta variety at each salt concentration compared to the values recorded for the other varieties at equivalent doses (Table 1).

A maximum of 44 μm was recorded for Ta in the control treatment, followed by decreases in the parameter as the salt concentration increased. These decreases were significant, by approximately 6% at S3 and 5% at S4 and S6 compared to the control. The minimum value was observed under S4 for Gl, which was significantly reduced by 17% compared to the corresponding control. The StoL assessment for Gr decreased significantly under all doses except for S5. Stomata lengths were significantly lower compared to the control (S1) for Gr, with differences in a range of around 4–7 μm under S2, S3, and S4 as well as under S6. The lowest impact on StoL was recorded for the Bo wheat variety, where the value range was narrow (39–41 μm) and the changes under all tested saline solutions were not significant compared to control (S1).

#### 2.1.3. The Effect of Wheat Variety × Salinity on Stomatal Width

The stomata width (StoW) changed significantly depending on the wheat variety and the interaction between wheat variety and saline solutions. The highest values were observed for the Gr variety tested under S3 and also for the Ta wheat tested with S6. These two values of approximately 25 μm showed significant increases compared to the values recorded in the corresponding control (S1), of approximately 10% for Gr and 8% for Ta (Table 2).

For the Gr wheat variety, StoW decreased significantly compared to the control by approximately 8% and 13% in plants tested with the S2 and S4 solutions. For Ta, the parameter value decreased significantly under S2, reducing StoW by approximately 6% compared to the control of the same variety. Regarding the Gl wheat variety, a single significant change in StoW compared to control was observed at S5. This variety showed the lowest values for this parameter and within the entire experiment the lowest variations due to salinity exposure. This record also marked the minimum of the entire set of values, decreasing by approximately 8% compared to the control. For the Bo variety, the stomata width reduced significantly compared to the control (S1) only under S5, recording a decrease of approximately 8%.

#### 2.1.4. The Effect of Wheat Variety × Salinity on Stomatal Aperture

The stomata aperture (StoAp) values differed significantly following the ANOVA test with a similar trend as StoL and StoW. The highest StoAp was recorded for the Bo wheat variety when tested with S4. This value represented a significant increase of approximately 17% compared to the control. (Table 3).

The minimum values for Bo showed significant decreases of approximately 31% at S3 and 28% at S5 compared to the control. For the Gl wheat variety, StoAp decreased significantly compared to control at three of the applied doses: S3, S5 and S6. The value recorded for this variety under S6 represented the minimum from the entire dataset, marking a decrease of approximately 31% compared to the control. For the Gr variety, the aperture values did not change significantly compared to the control, while for Ta, the application of the S3, S4 and S5 solutions significantly influenced StoAp and decreased the parameter values by 23%, 32% and 28% compared to the control.

#### 2.1.5. The Effect of Wheat Variety × Leaf Sampling Area × Salinity on Stomatal Length, Width and Aperture

Following the three-way ANOVA analysis, a significantly strong influence of variety on stomata length and width was observed (Table 4).

Variety also significantly (*p* < 0.05) influenced stomata pore aperture. The leaf sampling area strongly influenced stomata width and had a highly significant effect (*p* < 0.01) on stomata length values. The interaction between the tested saline solutions and leaf sampling area showed a highly significant variation (*p* < 0.01) in the stomata width parameter. On the other hand, the interaction between all three factors highlighted a significant (*p* < 0.05) variation only for stomata length.

#### 2.1.6. Interaction Between Stomatal Characteristics

**Figure 2:**
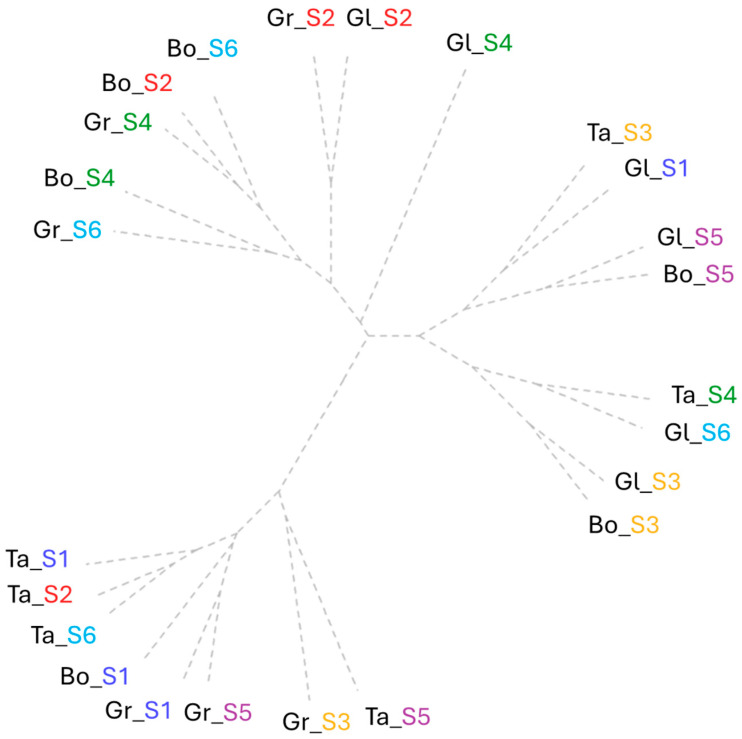
Cluster analysis of all stomata features assessed for each wheat variety tested (Bo—Boema, Gl—Glosa, Gr—Granny and Ta—Taisa wheat varieties) under all salinity concentrations (S1—control, S2—15 mM NaCl, S3—30 mM NaCl, S4—45 mM NaCl, S5—60 mM NaCl, and S6—75 mM NaCl).

The results obtained by clustering all the assessed stomata characteristics highlighted three major groups following the factor interaction wheat variety × saline dose. One of the criteria observed via clustering regarding stomatal density is the progressive decrease from cluster 1 to 3 at the leaf base and increases at the leaf tip. The general cluster trend showed medium cluster-based values of the parameters in cluster 1, minimum for cluster 2 and high for cluster 3 (Figure 2).

The first cluster includes variants with high SD at the base and middle leaf sampling area. Within the first cluster, three sub-groups were observed. Clockwise, starting with the Bo and Gr varieties, salinity levels S2, S4 and S6 showed medium values in the range of 33–40 for SD, 34–41 µm for stomata length at the leaf base and 2–3 µm for stomata aperture at the middle leaf sampling area. Further, the highest values (38–49) clustered Gr and Gl at S2 according to the DS parameter at the base and middle leaf sampling area.

The S4 salinity dose application showed the lowest values for stomata length in all leaf sampling areas together with aperture at the leaf base. The Bo wheat variety presented structural stability for S4 and S6 regarding DS values of 40 from leaf base sampling area. These two doses produced a slight reduction, with one unit for the parameters stomatal length (at the leaf tip) and for width (at the leaf middle and tip).

The second cluster represents a pair of two groups represented by 50% of the Gl wheat variety (S3, S4, and S5), Ta and Bo at S3. This was set by the cluster-based lowest values of the leaf middle SD (26–27 no/mm^2^), stomatal aperture and width at the leaf base and middle sampling areas. A group between the same salinity dose (S5) was formed from the varieties Gl and Bo following the same value of stomata (20 µm) width at the leaf base. This was completed with the osmotic regulation within the control and S5 for the Gl variety with the same trend for stomata width at the leaf base. The last group was formed by all the minimum values for stomata aperture in all leaf sampling areas at S3 and S6 for Gl and Bo, and also at S4 for Ta.

The last cluster includes 50% of the Ta variety, the control variant for Gr and Bo, and Gr tested under S3 and S5. These varieties were represented by a decrease in SD at the leaf base (27) and an increase in the same parameter at the leaf tip of 3 units. Although the values of stomata number were reduced at the leaf base, their length and width had higher average values compared to all variants. The first group is represented by Ta_S5 and Gr_S3, with a similar tendency of stomatal width values within the range of 22–27 µm in all leaf areas. The lowest SD values (in a range between 20 and 27 no/mm^2^) were at the leaf base groups of the control variants of Gr and Bo and with the Gr response to S5. This group was set also by the stomatal length at the leaf base, where the highest values obtained were 42–47 µm. The control (S1), the lowest salinity dose (S2) and the highest one (S6) grouped the Ta wheat variety by the stomatal length parameter assessed in the leaf middle area.

### 2.2. Wheat Relative Chlorophyll Content

Following the two-way ANOVA analysis, on six relative leaf chlorophyll (RCC) content assessments (C1–C6), the distribution of the values was influenced by the variety factor (*p* < 0.05). At the third assessment, 5 weeks after the beginning of the experiment, the salinity dose factor showed significant (*p* < 0.001) differences between the values of this parameter. The interaction between variety and saline doses did not highlight statistical differences, except for the C5 values (*p* < 0.05). Overall, a share of 54% of the varieties showed significant changes regarding RCC values compared to the corresponding control at least at one of the tested salt concentrations (Table 5).

Higher significant values compared to S1 were registered for Ta in all assessments, except C4. Overall, the same trend was observed for Gr at C3 and C4, for Gl at C2, C3, and C4, and for Bo at C1. Significantly lower values compared to S1 were observed only for Bo at C3 and for Gl at C4. At C1, no similar patterns were observed. The only significantly increased values compared to S1 were for Bo under S5, for Gr at S2, and for Ta at S4 and at the highest dose (S6). The following measurements (C2) highlight the S3 dose effect upon increased values of RCC for Gl and Ta. The next assessment (C3) emphasizes the twofold reduction produced by the sensitivity of Bo to the lowest-concentration solution (S2) compared to the control variant. Significantly higher values, in a range within 38–53 SPAD units, were observed for Gr at all applied salinity doses, more than twice compared to the control. The RCC for Gl and Ta increased under S4–S6, while S2 showed a reduction in the RRC value for Bo and an increase for Ta. At C4, the only significantly lower values compared to the control were seen for Gl at the S4 and S5 doses. Overall, the highest value of RCC (194 SPAD units) was recorded at C5 for Ta tested with S5. This value was five times higher compared to its corresponding control. At C6, the highest value, around two times higher than in S1, was observed for Gl at the highest salinity dose (S6). Extremely high sensitivity to the S6 treatment was observed at the final assessment, where the lowest RCC value, around 7 SPAD units, was registered for Bo.

**Figure 3:**
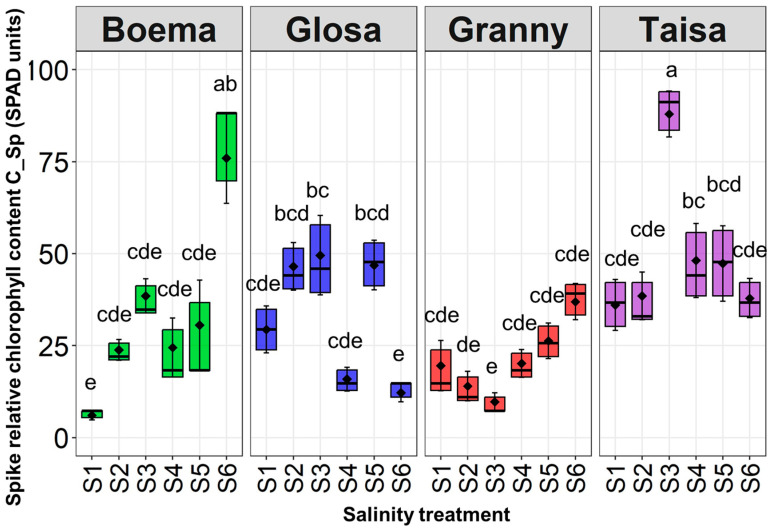
Spike relative chlorophyll content (C_Sp) registered to all wheat varieties and all saline treatments (S1—control, S2—15 mM NaCl, S3—30 mM NaCl, S4—45 mM NaCl, S5—60 mM NaCl, and S6—75 mM NaCl) at the end of the experiment. Distinct lowercase letters denoting statistical significance at *p* < 0.05 based on LSD test.

The highest spike relative chlorophyll content (C_Sp) value of 88 SPAD units was recorded for the Ta variety at S3 (Figure 3).

Testing S3 on Ta resulted in C_Sp at a twofold significant increase compared to the control value. Saline solutions (S2–S5) had no significant impact compared to the corresponding control for Gl and Gr varieties. The highest-concentration solution (S6) significantly increased C_Sp for Bo. This value was around 13 times higher compared to the control (Bo_S1), which represented the entire dataset minimum.

**Figure 4:**
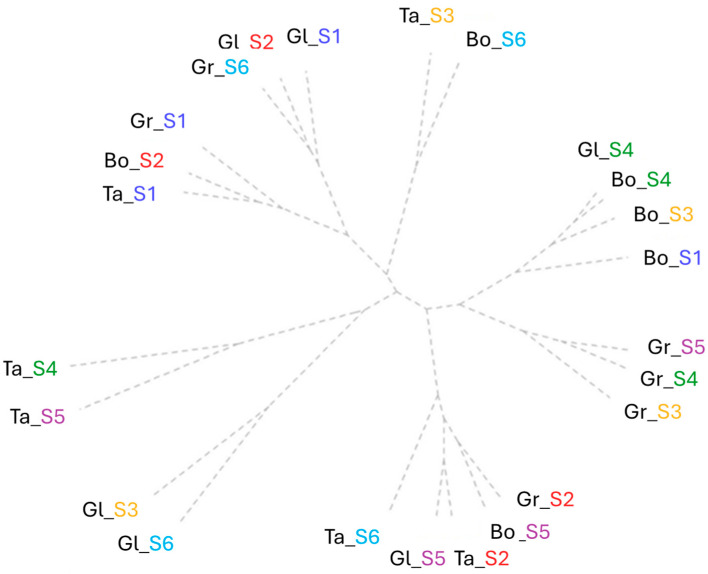
Cluster analysis of chlorophyll content assessments (C1—first leaves relative chlorophyll content assessment, C2—second leaves relative chlorophyll content assessment, C3—third leaves relative chlorophyll content assessment, C4—fourth leaves relative chlorophyll content assessment, C5—fifth leaves relative chlorophyll content assessment, C6—final leaves relative chlorophyll content assessment and C_Sp—spike relative chlorophyll content assessment) assessed for each wheat variety tested (Bo—Boema, Gl—Glosa, Gr—Granny and Ta—Taisa wheat varieties) under all salinity concentrations (S1—control, S2—15 mM NaCl, S3—30 mM NaCl, S4—45 mM NaCl, S5—60 mM NaCl, and S6—75 mM NaCl).

Relative chlorophyll content assessed at different times during the vegetation period grouped all varieties and doses into three principal clusters. The criteria for the first main group were based on the lowest values in a range between 20 and 39 SPAD units at C3 (Figure 4).

The minimum salt concentration solution (S2) influenced a reduction in RCC for Gl and Bo; S3 resulted in a low value (37 SPAD units) only for Ta, and the highest NaCl solution (S6) produced similar effects for Bo and Gr. The lowest value of 15 SPAD units in C1 was registered at the lowest salinity dosage (S2) for Bo, which was grouped with the control variants of Ta and Gr (15–24 SPAD units). Another group was represented by the highest values at C4 (151–171 SPAD units) and highlighted the close connection response in RCC between wheat varieties Gl (S1, S2) and Gr at the most concentrated solution (S6). A pair of variants, Ta_S3 and Bo_S6, induced the highest value on spike RCC (76–88 SPAD units) and the lowest wheat RCC range of 7–11 SPAD units at C4.

The second cluster shows the presence of Bo and Gr especially at S3, S4 and S5. Here, the medium values between 43 and 56 SPAD units were grouped for C3. The low value for C6 between 7 and 12 SPAD units grouped the Bo control (S1) and Bo_S3 with Gl and Bo tested under S4. Low values of spike chlorophyll content (10–26 SPAD units) and the first RCC assessment (C1) (25–28 SPAD units) were induced by the solutions within the range 30-60 mM NaCl in S3–S5 tested on Gr. A group effect induced similar responses of RCC in C1 (40–57 SPAD units) for the lowest salinity dose tested (S2) in Ta and Gr and for the high dose of 60 mM NaCl (S5) in Bo and Gl. The highest value obtained of 71 SPAD units in Ta wheat joined this group, with a similar effect at the highest tested dose (S6).

The third cluster comprised the highest values (between 42 and 64 SPAD units) for Gl and Ta. This was produced by the effect of S3 and S6 along with Gl on the RCC of C2. Linked to this group was the Ta RCC value under a 45 mM NaCl dose (S4) and the increased dose (S5), with the highest value of 194 SPAD units at C5.

**Figure 5:**
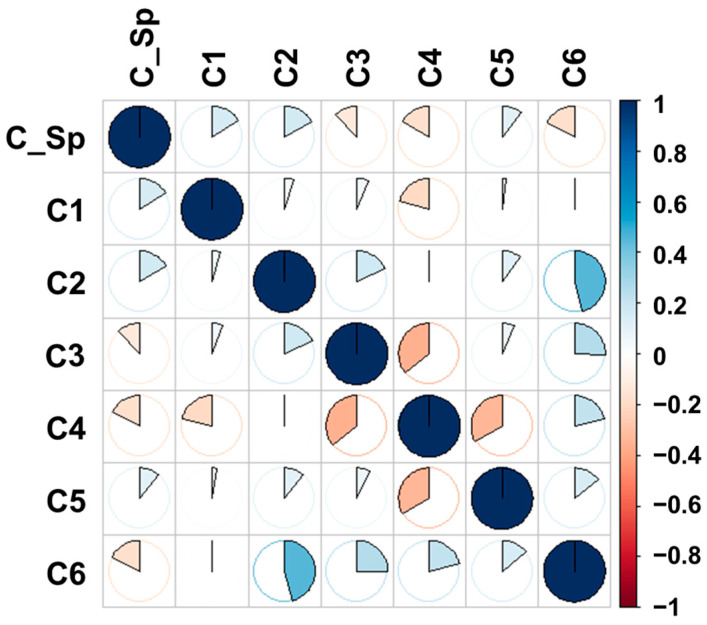
Relative chlorophyll content correlogram (C1—first leaves relative chlorophyll content assessment, C2—second leaves relative chlorophyll content assessment, C3—third leaves relative chlorophyll content assessment, C4—fourth leaves relative chlorophyll content assessment, C5—fifth leaves relative chlorophyll content assessment, C6—final leaves relative chlorophyll content assessment, and C_Sp—spike relative chlorophyll content assessment).

The C_Sp assessment took place during the grain filling phenophase, with the spike playing a major role in grain production. C_Sp shows weak to moderate correlations with the determinations in the other physiological stages evaluated (Figure 5).

The parameter shows low positive correlations when associated with the relative chlorophyll content of the leaves evaluated when the plants had a fully unfolded leaf (C1), two leaves (C2), and in the period prior to anthesis (C5). Also, the parameter values show a lack of dependence on the values of three (C3) and five fully unfolded leaves (C4) and from the moment of ripening (C6). The most obvious strong positive correlation was observed between the relative chlorophyll content of plants when they had only two leaves and at the time of ripening (C6). There is a stronger negative correlation between the parameter values when the plants had only three leaves and when they had five. This represents the plants’ reaction to stress over a longer period, with assessments carried out before and after vernalization, their attempt to adapt to saline presence in the nutrient area and climatic conditions. Another strong negative correlation was between the parameter values before anthesis (C5) and those during leaf growth and development (C4), marking an unstable trend in values as the plants approach maturity.

### 2.3. Wheat Morphological Parameters

#### 2.3.1. Plant Height

Plant height (PlantH), a morphological trait influenced by environmental conditions, mainly genetically determined, was significantly affected by the variety and salinity dose interaction and by variety factor alone. The Gl and Ta wheat varieties showed opposite trends in terms of this parameter evolution in the context of salt stress (Table 6).

In the control, the Gl wheat variety recorded a maximum PlantH of around 50 cm, and then the value decreased significantly with increasing salinity stress doses. This trend was evident at high doses in the range within 60–75 mM NaCl (S5, S6), where the values decreased by 21– 25%, respectively, from the maximum value recorded for the control.

The Ta wheat showed an opposite trend, and as the salinity stress doses increased, the PlantH values significantly increased. The tallest Ta wheat plants with a height of around 45 cm were observed at S4. This represented a share of 55% increase compared to the control. This trend indicated, from the point of view of this PlantH, an effective tolerance of Ta wheat to the tested salt doses. Throughout the increase in saline doses, plant height did not change significantly for the Gr and Bo wheat varieties.

#### 2.3.2. Spike and Awn Length

Spike length (S_L), a morphological trait influenced primarily by genetic factors, showed a distribution of values, based on two-way ANOVA analysis, with significant differences in parameters depending on the salt dose applied and the interaction between salt dose and variety (Table 7).

The longest spikes of around 9 cm were observed for the Ta variety, which, when subjected to the increased saline solution (S5), recorded a value approximately 26% higher than the control (S1). Spike length did not change significantly for Gr wheat throughout the increase in saline doses. A similar trend was observed for the Gl wheat variety. For Bo, spike length decreased significantly by 27% compared to the control only when tested with the 30 mM NaCl solution (S3).

Following the two-way ANOVA analysis, the awn length (A_L) value distribution indicates that the values were significantly determined by the different wheat varieties tested. The Bo wheat developed the overall longest awns under all tested saline solutions (Table 7). The highest parameter value for the Bo variety (5 cm) was recorded in the control (S1), and the values did not change significantly throughout the saline concentrations applied. A similar tendency was also observed for the Gl and Gr wheat varieties across all tested doses. A high impact of the lowest dose (S2) was registered for Ta, where the awn length decreased significantly compared to the control by approximately 42%.

#### 2.3.3. Spike Number

The productivity element spike number (Sp_no) obtained under salinity stress was affected, determined by the ANOVA test, by the different wheat varieties (*p* < 0.01). An Sp_no of approximately 27 was registered for Gr tested with the 30 mM NaCl solution (S3) (Table 8).

Furthermore, the use of saline solutions varying across the concentration range from minimum to maximum (S2–S6) did not result in significant changes for the Bo, Gl and Gr varieties. This trend did not apply for Ta, where increasing salinity doses were associated with a significantly higher number of spikes. The Ta wheat variety tested under the S2, S4 and S5 solutions showed significantly increased values of Sp_no of about eight, six and five times more compared to the control. The concentration of 30 mM NaCl (S3) and the highest salinity dose (S6) enhanced Sp_no (Ta), with values nine times higher than control.

#### 2.3.4. Heading Percentages

The maximum values of approximately 97% for the heading percentages (Spp) were associated with the increased salinity dose of 60 mM NaCl (S5) applied to the Gl wheat variety (Table 9).

Regarding Spp, two of the four varieties tested (Gl and Ta) recorded their lowest performance in the control group. Conversely, half of the tested varieties (Gl and Gr) achieved their higher heading percentage at S5. For the Bo, Gl and Gr wheat varieties, the tested saline dose (S2–S6) applications did not imply significant changes in values with increasing salt concentration. Despite a low heading percentage in the control of around 12% (S1), Ta exhibited an approximately sevenfold significant increase under the highest salinity dose (S6). Furthermore, the tested solutions within the range 15–60 mM NaCl (S2, S3, S4, and S5) improved Spp through significant increases, with approximately 6–7 times higher values than the control.

**Figure 6:**
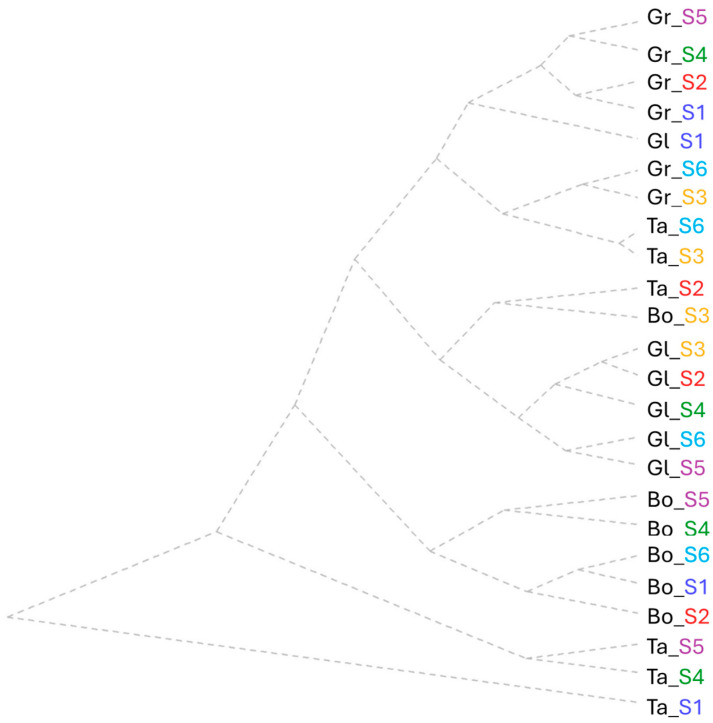
Cluster analysis of morphological parameters plant height (PlantH), spike (S_L) and awn length (A_L), spike number (Sp_no) and spike percentages (Spp) assessed for each wheat variety tested (Bo—Boema, Gl—Glosa, Gr—Granny and Ta—Taisa wheat varieties) under all salinity concentrations (S1—control, S2—15 mM NaCl, S3—30 mM NaCl, S4—45 mM NaCl, S5—60 mM NaCl, and S6—75 mM NaCl).

The cluster of all morphological parameters assessed could be seen in five main groups by the variety in different development and productivity. The first group is represented mainly by Gr and the next one by Gl, followed by Bo, then by Ta. The first cluster was set by higher plant height values (43–46 cm) obtained for Gr at all salinity doses, along with the highest parameter value of 50 cm for the control in Gl and with Ta at S3 and S6 (Figure 6).

Two other parameters, spike number and percentages, linked this cluster, with the highest values connected with Gr wheat and previously mentioned doses.

The second cluster was emphasized because of the highest values of Spp seen at S3, S4, and S5 doses in a range between 91 and 97% within the highest value at the latter dose at Gl. The lowest saline solution (S2) produced the lowest spike (6 cm) and awn length (2 cm) of Ta. This cluster also links the effect of the S2–S3 solutions transposed in medium values obtained for plant height (Ta_S2 and Bo_S3) and spike number (Gl_S2 and S3).

The third cluster emphasized Bo at all doses except S3 grouped by the high value of shoot number (28–29) and highest values for shoot (8–9 cm) and awn length (5 cm) across the S4–S6 salinity levels and the control.

The following cluster was represented by the Ta variety, with the highest values (9 cm) for shoot length after being tested with S5 and medium values for plant height (45 cm) and awn length (4 cm) by the effect of the moderate dose of 45 mM NaCl (S4).

The last cluster comprises the lowest values of plants height (29 cm), spike number (20) and heading percentage (12%) from the Ta control treatment.

### 2.4. Wheat Biomass

#### 2.4.1. Shoot and Spike Fresh Biomass

**Figure 7:**
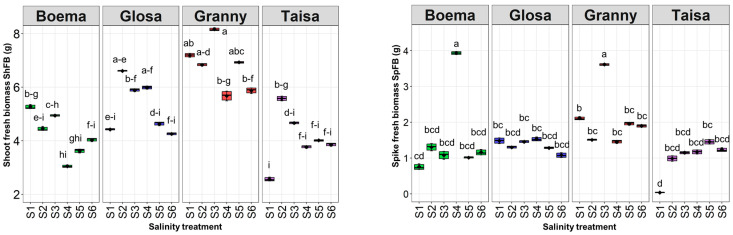
Shoot (ShFB) and spike (SpFB) fresh biomass in g of all wheat varieties depending on the saline solution concentrations: S1—control, S2—15 mM NaCl, S3—30 mM NaCl, S4—45 mM NaCl, S5—60 mM NaCl, and S6—75 mM NaCl at the end of the experiment. Distinct lowercase letters denoting statistical significance at *p* < 0.05 based on the LSD test.

Only half of the tested varieties’ shoot fresh biomass (ShFB) was significantly changed compared to the corresponding control after applying the saline solutions (Bo and Ta) (Figure 7).

A maximum ShFB of around 8 g was observed for the Gr variety at S3. The overall minimum value was obtained at Ta_S1, around four times lower than the maximum registered.

All saline solutions applied did not lead to significant decreases in ShFB values compared with the corresponding control for the Gl and Gr varieties. A minimum of around 3.05 g ShFB for Bo was recorded under the moderate dose of 45 mM NaCl (S4). This value represents a significant decrease of around 42% compared to the control (Bo_S4).

Furthermore, the lowest NaCl solution (S2) application had a stimulating effect, with a value more than double compared to the control for Ta wheat.

A maximum value of around 4 g for spike fresh biomass (SpFB) was registered for the Bo variety tested with the moderate saline solution (S4) (Figure 7). This value represented a significant increase, which was five times higher compared to the control. Another significant increase of 71% was observed for the Gr variety SpFB (3.61 g) tested with S3 compared to the corresponding control. The increased salinity dose (S5) applied on Ta wheat was associated with a significant increase in the SpFB value (1.45 g) compared to the corresponding control (Ta_S1), which was the entire dataset minimum. The saline doses applied (S2–S5) had no significant impact on SpFB.

#### 2.4.2. Shoot and Spike Dry Biomass

**Figure 8:**
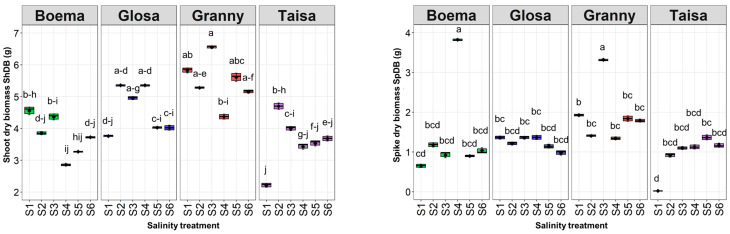
Shoot (ShDB) and spike (SpDB) dry biomass in g of all wheat varieties depending on the saline solution concentrations: S1—control, S2—15 mM NaCl, S3—30 mM NaCl, S4—45 mM NaCl, S5—60 mM NaCl, and S6—75 mM NaCl. Distinct lowercase letters denoting statistical significance at *p* < 0.05 based on the LSD test.

Carbon allocation maximum values, seen in shoot dry biomass (ShDB), were observed for Gr at S3, S1 and S5 (Figure 8).

The highest value was around 7 g, under the 30 mM NaCl solution (Gr_S3). Higher sensitivity was observed in Ta without a salinity effect (S1), where the ShDB values were the lowest (2.21 g). However, testing S2 and S3 led to significant increases in Ta carbon allocation, approximately 112% and 81% compared to the control. Testing solutions in the concentration range within 15–45 mM NaCl (S2–S4) had opposite effects for the Bo and Ta wheat varieties. Therefore, Bo ShDB values significantly decreased by around 37% under the moderate saline solution (S4). The ShDB of the Gl variety did not have any significant changes across all the tested saline doses (Figure 8).

The spike dry biomass SpDB followed a trend similar to that observed for the SpFB. An SpDB maximum of around 4 g was recorded for Bo under the 45 mM NaCl solution (S4). This value was significantly increased by about six times higher compared to the control (S1). Another significant increase in SpFB, with a value of almost two times compared to the control, was observed for the Gr wheat variety tested with the 30 mM NaCl solution (S3). The increased saline solution (S5) stimulated SpDB for Ta, with a 1.35 g difference compared to the control (Figure 8).

#### 2.4.3. Shoot and Spike Moisture Content

**Figure 9:**
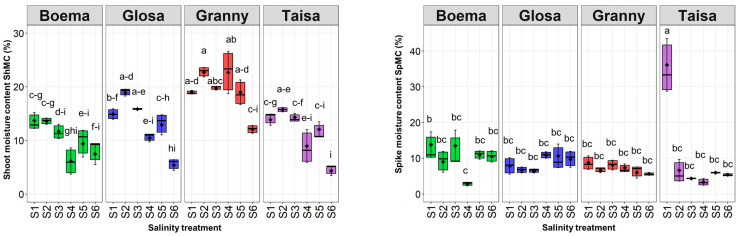
Shoot moisture content (ShMC) and spike moisture content (SpMC) of all wheat varieties depending on the saline solution concentration: S1—control, S2—15 mM NaCl, S3—30 mM NaCl, S4—45 mM NaCl, S5—60 mM NaCl, and S6—75 mM NaCl. Note: Two-way ANOVA between V—different wheat varieties tested, S—different saline solutions and V:S—interaction for ShMC V (F = 3.73, *p* = 0.057); S (F = 24.21, *p* < 0.001); V:S (F = 0.02, *p* = 0.878) and SpMC V (F = 0.02, *p* = 0.891); S (F = 7.16, *p* = 0.009); and V:S (F = 9.92, *p* = 0.002). Distinct lowercase letters denoting statistical significance at *p* < 0.05 based on the LSD test.

Stem moisture content (MC) exhibited a similar trend across three of the four tested varieties. Gl, Gr, and Ta recorded the highest ShMC levels between 16 and 23% at the lowest salinity dose (S2). Subsequently, the parameter performance declined, reaching minimum values (4–12%) at the highest concentration solution (S6), which were significant for Gl and Ta (4–5%) (Figure 9). The Bo wheat variety recorded the lowest values of ShMC for almost all salinity doses tested, without significant decreases. The minimum dataset of ShMC was associated with the S6 application for the Ta wheat variety, with a significant decrease of around 10% compared to the control. A similar significant decrease due to the S6 dose was observed for the Gl wheat variety. A maximum ShMC of around 23% was seen for the Gr wheat tested with the lowest salinity dose (S2).

The tested saline solutions (S2–S6) did not significantly change the SpMC for half of the tested wheat varieties (Gr and Gl) (Figure 9). A maximum SpMC of around 36% was observed for Ta from the control. This value was significantly reduced with increasing salinity doses (S2–S6) by 29% for S2, 32% for S3, 33% for S4, 30% for S5 and 31% for S6. Another significant reduction of around 11% compared to the control was observed for the Bo variety tested with the moderate saline solution (S4).

**Figure 10:**
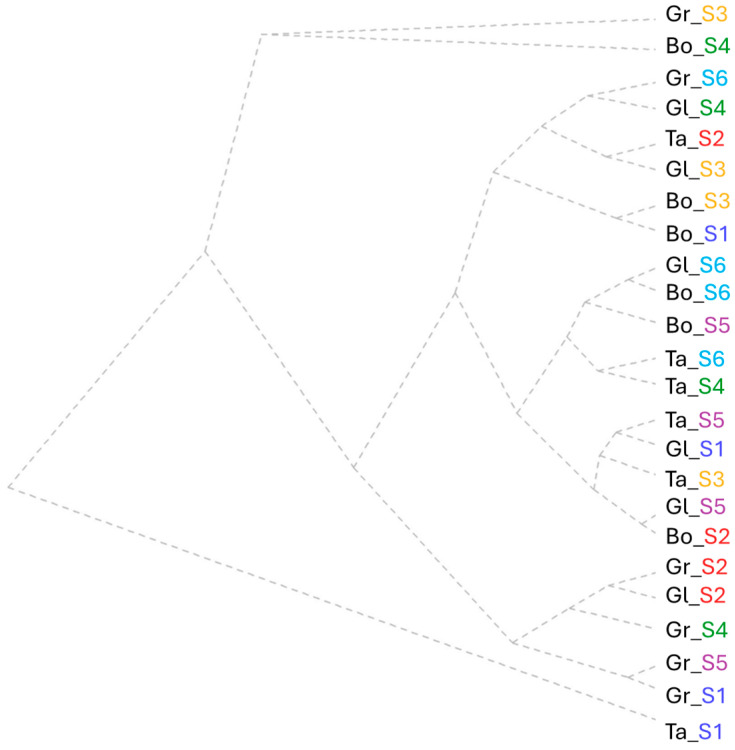
Cluster analysis of fresh biomass for shoots (ShFB) and spikes (SpFB), dry biomass for shoots (ShDB) and spikes (SpDB), and moisture content for shoots (ShMC) and spikes (SpMC) assessed for each wheat variety tested (Bo—Boema, Gl—Glosa, Gr—Granny and Ta—Taisa wheat varieties) under all salinity concentrations (S1—control, S2—15 mM NaCl, S3—30 mM NaCl, S4—45 mM NaCl, S5—60 mM NaCl, and S6—75 mM NaCl).

The biomass assessed and moisture content grouped the variety and salinity doses tested into three major clusters. One is defined by S3, with the effect of the most performant wheat variety being Gr because of the highest values obtained for shoot fresh biomass (8 g), dry biomass (7 g) and spike fresh biomass (4 g). This was linked by an increased dose in the moderate saline solution (S4) having an effect upon Bo following the same trend for spike fresh and dry biomass (Figure 10).

The second large cluster was defined by most varieties and doses. Three other groups were observed. Primarily, the medium values of the parameter shoot fresh (5–6 g) and dry biomass (4–5 g) connected Bo control, Ta_ S2, Gl_S4, Gr_S6, and Bo with Gl after S3. Second, Ta represents a bridge group, which links minimum values for shoot fresh (4 g) and dry biomass (3–4 g) with the lowest value for spike moisture. The last group ranked the highest values of shoot moisture between 19 and 23% in Gr with the lowest and moderate salinity doses (S2–S4). This group also emphasizes the overall increased value for shoot fresh and dry biomass even at the increased dose (S5) being represented mainly by the Gr variety.

The control treatment of Ta is the only assignee of the third cluster. For this variant, a late development was observed due to which the highest values were recorded for spike moisture (36%) and the lowest values for shoot and spike fresh and dry biomass.

### 2.5. Morpho-Physiological Parameter Interactions

**Figure 11:**
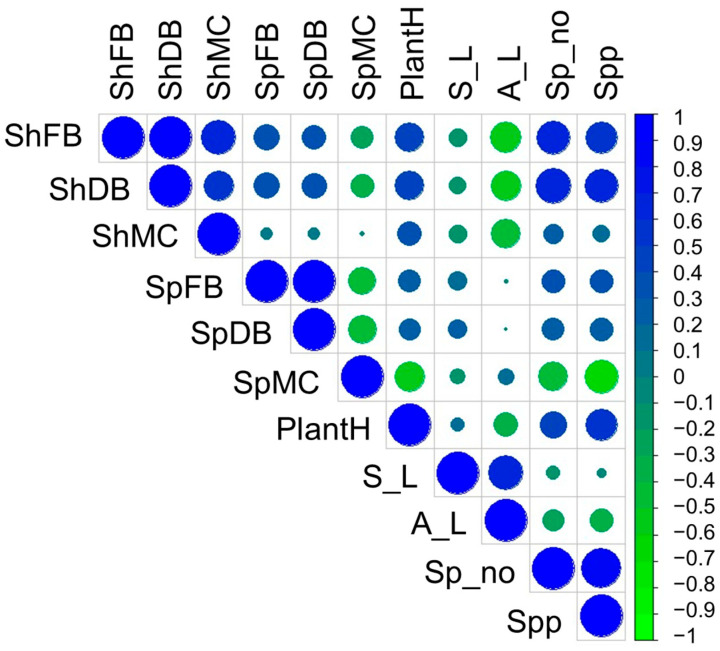
Correlogram between plant height (PlantH), spike (S_L) and awn (A_L) length, shoot (ShFB) and spike (SpFB) fresh biomass, shoot (ShDB) and spike (SpDB) dry biomass, shoot (ShMC) and spike (SpMC) moisture content, number of spikes (Sp_no) and heading percentage (Spp) assessed at the end of the experiment.

Green and dry biomass, as well as moisture content in shoots, showed the strongest positive correlations within the group of parameters considered (Figure 11). This indicated proportional variation in the parameters.

The same parameters evaluated for shoots did not show similar dynamics in spikes. Although spike biomasses are strongly correlated, the association of the values of the two parameters with the moisture content in spike presents average negative correlations. This parameter evaluated on the spike shows generally negative correlations with most variables and a weak correlation when associated with the same parameter evaluated for the shoots. The green and dry biomass of the shoots are strongly correlated with those of the spikes. This reflects the fact that good shoot development can be associated with spike development. Plant height is strongly correlated with all parameters except spike moisture content. Spike and awn length, being strongly genetically determined, showed varied distributions of values. These two parameters showed strong or moderately negative correlations with most parameters. Spike length, on the other hand, showed moderately positive correlations with the morphological parameters of the spike.

## 3. Discussion

One of the most significant abiotic stress, salinity, suppresses agricultural yields worldwide, while crops exhibit divergent patterns responses in terms of ecophysiological adaptations [27]. Overall plant native adaptations to climate and salinity could be visible following morphological representative characteristics and throughout metabolism changes, which impact some physiological parameters [28]. Salinity stress for wheat is usually mediated by a complex salt tolerance mechanism throughout stomatal alterations, osmotic regulation, ion exclusion, antioxidant defense mechanisms and hormonal balance [29,30].

**Figure 12:**
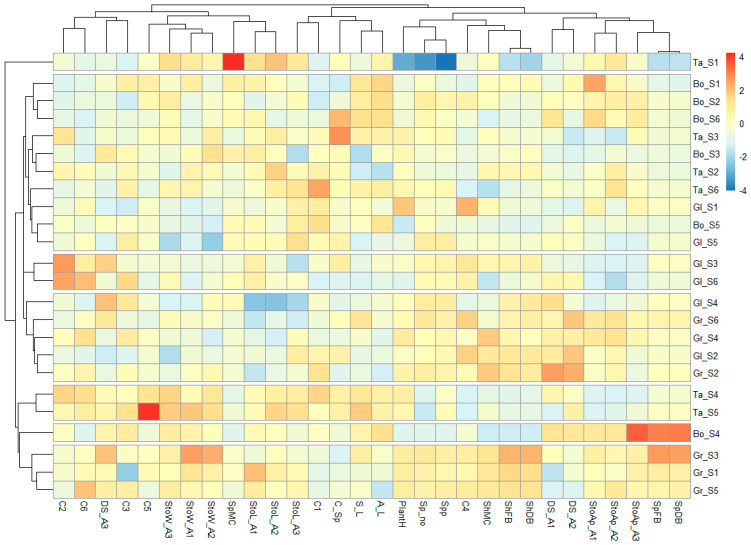
Heatmap representation of variety × salinity dose interaction for all assessed parameters.

The detailed response of varieties to salinity stress showed multiple physiological hotspots of each variety (Figure 12) via Z-score reaction. Two cases (Ta_S1 and Bo_S4) had a set of parameters that individualized them within the entire database. Taisa scored low values for plant height and spike number and percent but high values for spike moisture, while Boema scored high values for spike biomass and stomatal aperture. Salinity levels S1, S3 and S5 produced a convergent reaction in Granny but with specific high–low scores for parameters, which grouped them in the lower part of the heatmap. The S3 dose sustains high values for stomatal width and density (A3) and spike and shoot biomass, but S5 acted as a threshold, decreasing stomatal density in base and middle leaf area. Taisa (S4 and S5) shows a divergent trend of biomass reduction coupled with an increase in stomatal and chlorophyll traits, a phenomenon consistent with the chlorophyll concentration effect due to leaf area reduction. The elevated SPAD values observed in Taisa represent an example of morpho-physiological adaptation to salinity; in recent studies it was documented that salt stress can induce a reduction in leaf area and increase the thickness [31,32]. The group defined by Gr_S2–Gl_S4 is characterized by higher values for biomass and stomatal aperture but lower values for stomatal length and varied chlorophyll throughout the vegetation period. Glosa’s reaction to S3 and S6 shows higher values for C2 and C6 chlorophyll but slightly lower values for the rest of the parameters. One high group (Gl_D5–Bo_D1) showed divergent low–high hotspots. The S5 dose reduced stomatal width for Glosa and Boema and maintained the other parameters within a medium–low range. Taisa (S2 and S6) scored higher values for stomatal length, while at S3 it reached high values for spike chlorophyll. Boema’s reaction to S6 shared similar reactions as in the control and the lowest salinity (S1 and S2), with lower chlorophyll and medium biomass.

**Figure 13:**
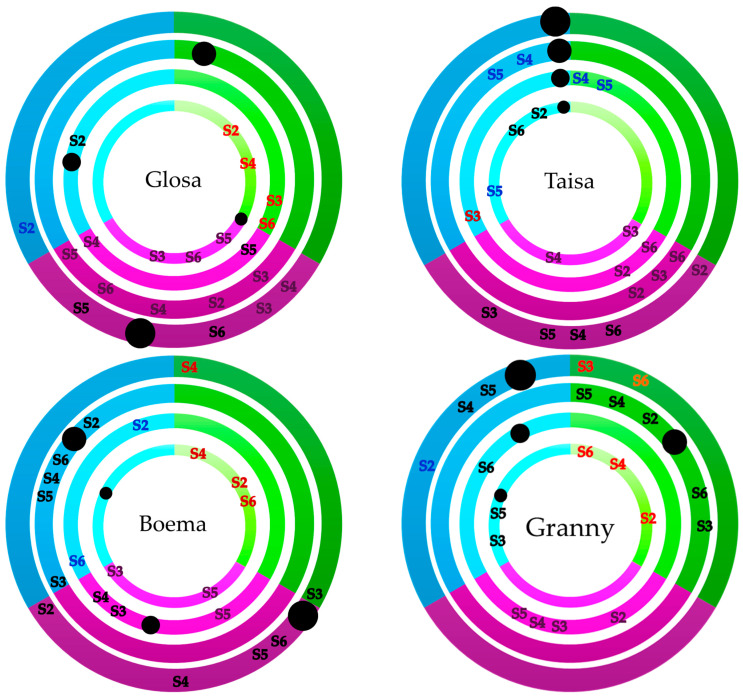
Pattern modeled of all four wheat varieties tested with all doses ranked by individual parameter clusters. From inside out the first line represents stomatal features, the second chlorophyll values, the third visual growth and development parameters and the fourth the result of all three seen in biomass (black dots—control, S2—15 mM NaCl, S3—30 mM NaCl, S4—45 mM NaCl, S5—60 mM NaCl, and S6—75 mM NaCl; green—high values, purple—medium values and blue—low values; different colors of salinity doses abbreviations represent different associations in a group within the clusters).

Several negative effects produced by the salinity stress were categorized in four joint classes based on our results. Two were physiological parameters, stomatal features and relative chlorophyll content, and two morphological parameters, productivity seen in biomass allocation and growth features (Figure 13). Salinity stress did not act uniformly within different doses on the four wheat varieties regarding the morpho-physiological parameters assessed.

The multi-layered sunburst plot was designed to emphasize individual components throughout an integrated result within the four established joint classes. Using the inside–out synthesis model logic, the three inner rings represent stomatal features, chlorophyll values and visual parameters like plant height and spike length, ending with spike percentage acting as a composite index for the fourth ring. This integrated physiological mapping follows a hierarchical synthesis model where the four joint classes represent the cumulative signature of the variety to define the observed stress-tolerance level. The results demonstrated that stomatal density and aperture can be used as predictive parameters for vigor screening of the varieties. The mapping procedure highlights how the selection for smaller but denser stomata can optimize the water use efficiency under salinity stress, providing a more detailed analysis of variety reaction than the use of morphological characteristics alone.

One evident aspect can be highlighted by the control values of all of Taisa’s parameters. This suggests a unified physiological signature indicating a balanced metabolic outcome. Taisa and Boema demonstrate their maximum cumulative response at 45–60 mM through the highest chlorophyll values.

Taisa and Granny show a more specialized physiological focus and dominant performance trait.

Maximum biomass values were not registered for Taisa. Average values of the visual parameters combined with medium values of stomatal features exposed to 30 mM NaCl and with medium chlorophyll values exposed to 75 mM NaCl were transposed into medium biomass values. Only the doses 45 and 60 mM NaCl resulted in maximum values for chlorophyll, suggesting Taisa specialization within this physiological parameter that is detrimental to visual morphologic parameters. For the lowest tested dose, the pattern highlights low values for stomata and average values for chlorophyll and visual parameters; therefore, biomass was ranked in the medium class, which again emphasizes that stomata do not influence carbon allocation.

Granny showed a reinforced response; while the inner rings overlap within the same sector, the variety is prioritizing that specific metabolic pathway. A more sensitive but rapid adjustment was happening under 30 mM compared to the other cultivars. At this dose, the values of all visual parameters are strongly influencing high carbon allocation in biomass. The doses 15, 45 and 75 mM NaCl emphasized high values for stomata features and visual parameters, which resulted in low values for biomass and high values for the highest dose. Despite the fact that the highest values of visual parameters were seen for 60 mM, the low stomatal values and decreased values of chlorophyll resulted in biomass decline.

Glosa indicates that the combined influence reached a critical threshold, representing a significant stress-response marker. Most of the doses highlight medium trends for all parameters, seen in medium biomass values in the end. A significant cumulative weight at the higher concentration of 75 mM reflects a transition from adaptive adjustment to a robust stress-response mechanism given by chlorophyll content and seen in medium biomass allocation. A similar tendency was seen for 30 mM NaCl for the same parameter. Stomatal features were increased by counteracting the 15 and 45 mM salinity dose. The 45 mM threshold represents the point where Glosa effectively activates Na^+^ compartmentalization into the vacuole, maintaining cytosolic K+ levels for the guard cell function. The lowest salinity dose determined low values for chlorophyll, seen also in biomass.

Boema demonstrates a decentralized reaction, reflecting a broad distribution in terms of biomass. The maximum cumulative response for this variety was at 45 mM through the highest stomatal values proportional with biomass. This confirms that moderate salinity determines an osmotic gradient and maximizes turgor-driven processes without reaching the phytotoxic range. Stomatal features were also satisfactory at the lowest and highest tested doses. The negative visible effect of growth parameters was clear at all doses, with two interconnected points with chlorophyll content degradation at 15/75 mM NaCl. Biomass with average values in the center chain was influenced by the 60 mM dose.

The growth and development of plants from the Poaceae family, such as wheat, ryegrass and rice, are inhibited by lower concentrations than 100 mM NaCl [33,34]. Within the tested wheat varieties, a uniform reaction regarding stomatal density was observed for Bo and Ta at the S1–S5 doses. The other two varieties, Granny and Glosa, had a plant-specific reaction that was seen in the large range of the SD reaction [35]. Overall, the observed response highlights a clear separation between two levels of salinity stress: at 15–45 mM the varieties show an osmotic adjustment that is visible in the maintenance of stomatal aperture, while at 60–75 mM the ionic toxicity is visible in the metabolic decline and biomass reduction.

The stomatal density trend highlights increased values due to the moderate NaCl dose (S4). This could be explained by the salinity-stimulating effect on wheat, connected with homeostasis and breaking the stress by counteracting salinity [30]. The biphasic stomatal response is consistent with findings in related genotypes where moderate stress determines a compensatory increase in stomatal density as a form of phenotypic plasticity [36], and the maximum aperture recorded at 45 mM suggests a critical point where the accumulation of Na+ in the guard cells acts to lower osmotic potential and facilitate water influx to maintain turgor [31]. The observation of smaller but denser stomata represents an adaptive strategy to optimize the plant water use efficiency. Based on the findings of Hasanuzzaman et al. [32], smaller stomata possess faster opening/closing dynamics, allowing the plant to maintain a minimum photosynthetic rate while avoiding water loss through excessive transpiration. This mechanism relies on the signaling of abscisic acid that forces a defensive stomatal closure after the salt stress exceeds the plant’s threshold [37]. Stomatal-based salinity stress resilience was observed for the Granny variety, and we classified it as highly tolerant. On the contrary, rice plants exposed to 20 and 50 mM NaCl with reduced stomatal density highlighted improved performance [38]. The observed differences underline the importance of genetic background in salinity response. An average tolerance was observed for Glosa. This variety represents an intensively cropped variety in Romania because of farmers’ traditions and good experiences with its crop yield [39,40,41]. The maintenance of the balance between chlorophyll content and stomatal opening demonstrates the capacity to manage salt stress, an observation that sustains the early selection for physiological vigor in breeding along with targeting the development of varieties adapted to salt stress. The Boema variety showed a low tolerance, perhaps due to a low stomatal-based salinity stress resistance [38]. Taisa, a newly obtained facultative variety with late-maturing characteristics, was created to resist lodging stress [42,43] and proved to be the most sensitive to the tested saline concentrations, in accordance with the literature.

A specific reaction of stomatal length was observed in the Taisa variety, where the cells were the longest at all salinity doses. This could be due to genetic heritage obtained from amelioration [29] sustained by the maximum value registered in the control treatment. The two–three steps of increasing salinity doses produced a slight reduction because of low stress acceptance throughout low tolerance [29].

The cell width observed for Gr due to the 30 mM NaCl solution was the highest due to turgor pressure and the osmotic gradient, which allow the guard cells to fill with water because the water potential outside the cell is higher compared to the water potential inside the stomata [44]. The salinity dose of 15 mM NaCl did not sufficiently decrease the osmotic potential, and the moderate turgor pressure lead to narrower stomatal width [38]. When the salinity dose was increased three times, osmotic adjustment was observed after guard cell wall deformation due to elevated hydrostatic pressure and ion homeostasis in the cytosol, which resulted in a visible stomatal width [30]. Stomatal aperture had the highest values under the moderate saline solution of 45 mM NaCl, with Boema having the most active physiological redundant metabolism.

The chlorophyll contents of Taisa, Granny and Glosa showed resilience to salinity (45 mM NaCl). This suggests a superior antioxidant system and a better ion exclusion at the root level, preventing Cl^−^ toxicity in the leaves. From the first measurements, the Bo variety was the most sensitive, with lower values for chlorophyll. In time, chlorophyll-based salinity stress resilience observed in Glosa and Boema had a deficit in counteracting the stress effects. Salinity stress negatively affects the leaf greenness [28,45] of Boema because of its genetic nature, being a very early variety that is resistant to winter and heat [46], however, not to the highest salinity dose. Chlorophyll content is primarily involved in photosynthesis and also contributes to protection against oxidative stress along other metabolic pathways [47]. The physiological transitions observed at higher salinity levels in soil are closely linked to the activation of a specific ionic homeostasis mechanism, likely triggering the salt overlay sensitive signaling pathway. In tolerant varieties, like Glosa, this pathway facilitates sodium efflux from the cytosol back to the apoplast [48,49]. Furthermore, the resilience of Glosa at S4 suggests a superior tissue tolerance, characterized by efficient vacuolar sequestration of Na+. This process reduces cytosolic toxicity, maintaining cellular turgor without degrading chlorophyll, which was visible in the concentration effect observed [49,50,51].

The visual responses of salinity were assessed by morphological parameters. Proportional with the physiological parameters, Granny registered the maximum plant height values (60 and 75 mM), spike length (30 and 75 mM) and spike number (30 mM). Yield component parameters such as spike length and biomass have also been shown to be affected by salinity stress [45]. If there are salts in soil, keeping the level at 45 mM NaCl would benefit Taisa and Granny growth. A clear indication of salinity stress could be seen by a reduction in shoot growth, which results in changes to the biomass allocation between roots and shoots [45]. The counteracting effect of salinity stress (60 mM) in terms of heading percentage was shown for Glosa. Other results obtained when Taisa was assessed without salinity stress showed increased morphological parameter values and phenotypic expression due to autumn sowing [52].

Salinity presence enhanced cell osmosis, and the only significant positive response in fresh biomass was observed at the lowest dose for Taisa. The highest sensitivity for this parameter was at 45 mM for Boema; however, the variety tried to compensate by accelerating the allocation of carbon to the spike fresh and dry biomass. The same dose increased the Boema spike fresh and dry biomass, which was the highest, followed by Granny. These results are consistent with other studies where Boema biomass was compared to Glosa [53]. Under the 30 mM dose, Granny accumulated the highest dry biomass. At the dose of 45 mM for all wheat varieties, a reduction was observed for this parameter. The correlation established between microscopic parameters and biomass accumulation demonstrates that maintaining stomatal functionality at 45 mM (S4) is responsible for the stability of morphological parameters. Sensitive varieties, such as Granny, exhibit a biomass reduction correlated with a decrease in stomatal density, while tolerant varieties have a compensatory mechanism in the increased stomatal density. These phenomena confirm that stomatal assessment can serve as a tool for predicting biomass under saline stress and that it is an effective method for germplasm evaluation.

The shoot moisture content emphasizes the dehydration level, starting with the first dose tested, 15 mM NaCl, up to the highest concentration applied. This means that tolerance levels are unique features with different paths for each one of the varieties tested. Taisa, the new late variety, succeeded in keeping a high spike moisture content.

The experimental design enabled the analysis of both morphological and physiological parameters along an entire vegetation cycle, allowing the isolation of salinity effects from field environmental variability. Despite the limitations of the study, our findings provide a framework for subsequent multi-year trials focused on the tolerant genotypes (as Glosa and Taisa), confirming the validity of using microscopic parameters as indicators for early stage selection. These data refine our understanding of the different wheat varieties’ morpho-physiological implications due to osmotic adaptation. The multi-layered data reveals that the final physiological state of the plant is not always a sum of its components but a weighted synthesis. While individual metrics of the first three rings show minor fluctuations, the outside ring provides the final metabolic osmotic fingerprint for each tested variety under salinity stress.

## 4. Materials and Methods

A field-pot mesocosm experiment was set up in mid-October under field conditions. The experimental design consisted of four varieties and six salinity doses in five replicates. The first step was to place 22 cm h, 24 cm diameter vegetation pots on the ground. These were filled with a volume of 10^−2^ m^3^ clay–loam soil and at a bulk density of 1.3 Mg/m^−3^ from the USAMV Cluj-Napoca agrobotanical garden. Soil humidity was adjusted to 75% of field capacity, and the chemical properties along with electrical conductivity (EC) were assessed for control soil at the beginning and at the end to observe the natural salinization (63–65 mS) and also for all applied salinity treatments, which were overall in a range between 70 and 88 mS [54]. To prevent the NaCl solutions tested to simulate salt stress from leaching into the soil, the vegetation pots were isolated with a resistant low-density polyethylene film. To ensure uniform exposure and avoid salt shock, treatments were calculated relative to the mass of soil in each pot, ensuring a homogeneous distribution of osmotic potential in the root zone. Although the electrical conductivity (EC) of the soil can vary depending on evapotranspiration, the insulation of the pots with foil had a dual role: preventing overheating of the root system and limiting concentration fluctuations through direct evaporation at the surface. This methodological rigor allows us to attribute the observed physiological variations directly to the applied NaCl gradient, minimizing the interference of secondary environmental factors. There were no irrigation conditions except for this first application of 500 mL from each salinity dose applied. Climatic conditions were monitored for the entire vegetation season with a Davis Vantage Pro2 meteorological station (Hayward, CA, USA), which was placed in the experimental field (data published in [36]).

The stress applied consisted of a control and a series of NaCl solutions in gradually increasing concentrations. Thus, the doses were: control (S1), water only, the first salt concentration of 15 mM NaCl (S2), and then 30 mM NaCl (S3), 45 mM NaCl (S4), 60 mM NaCl (S5), and the highest dose of 75 mM NaCl (S6) in five replicates. A one-time application was used at the soil surface before sowing the seeds at the beginning of the experiment.

### 4.1. Wheat Varieties

**Figure A2:**
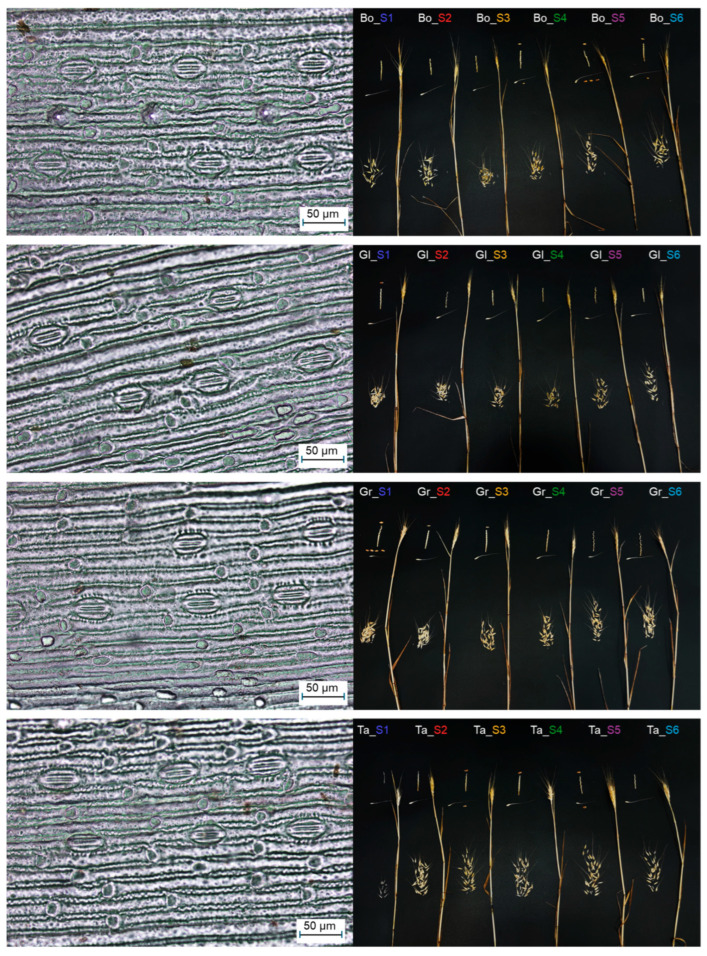
Phenotypic features of all varieties and all salinity doses and stomata.

The biological material tested consisted of seeds from four varieties of wheat (Figure A2). These were procured by ARDS Turda. The varieties tested were as follows: Boema and Glosa, autumn varieties; Granny, spring wheat; and an optional variety of wheat, Taisa. A number of 30 seeds was sown in the fall to evaluate their comparative behavior under the same saline and environmental stress conditions. Boema is a variety registered in 2000 [55], recommended in winter wheat improvement programs due to its special agronomic characteristics [26]. The medium–early growth period determines high gluten quality. It is adapted to various climatic and soil conditions and resistant to winter, falling and drought. The early-maturity wheat variety Glosa was produced by the National Institute for Agricultural Research and Development Fundulea and registered in 2005 [55], with high ecological plasticity, increased heat tolerance and drought resistance. This winter wheat is one of the most widely grown varieties in southern Romania [56]. Granny is a medium–late spring wheat variety [57] registered in the Czech Republic in 2004, originating from the crossbreeding of a spring wheat with a winter wheat. It is distinguished by its high adaptability to various ecophysiological conditions [58], average lodging resistance and high yielding potential. Taisa is a modern variety, a recent facultative wheat with a late-maturity growth period optimized for high nutrient and water use efficiency [42].

### 4.2. Applied Methods

During the experiment, the relative chlorophyll content of the leaves was evaluated at certain BBCH (Biologische Bundesanstalt, Bundessortenamt, and CHemische Industrie) stages using a non-destructive method. These assessments performed during all vegetation growth seasons at key phenophases for wheat (C1—one true leaf, C2—two fully developed leaves, C3—three fully developed leaves, C4—five fully developed leaves, C5—anthesis beginning, and C6—senescence) were performed using an MC-100 chlorophyll meter manufactured by Apogee Instruments (North Logan, UT, USA), which expresses the parameter values in SPAD units. The same parameter was also assessed on wheat spikes (CSp) before the end of the experiment. Consistency in the measurements was assured by the selected hour analysis range between 9:00 and 11:00.

For the stomata parameter analysis, leaf imprints were sampled at the experimental end from the abaxial surface of wheat leaves at the base, middle, and tip of the flag leaf on a sunny day between 9 and 11 AM. The non-destructive nail polish impression method was used. A layer of nail varnish was applied to the back of a leaf. Once it dried, the nail polish films were removed using transparent adhesive tape and applied to microscope slides. Nine microscopic fields were assessed per each collected sample. The obtained imprints were assessed under an Olympus CX 43 microscope (Tokyo, Japan) with 40× magnification to identify the number of stomata. This parameter was used to determine the stomatal density (number of stomata/mm^2^). The imprints were assessed with PROMICAM PRO Digital Camera 8MP (Prague, Czech Republic), the images were captured, and the stomata number was counted. Then, in the calibrated software QuickPHOTO CAMERA 3.2 (Prague, Czech Republic), the images served to obtain the stomata aperture, lengths, and widths in µm using the “Measurements” button. All data was automatically imported in an Excel file into the microscope program then exported for further statistical analysis.

At the end of the experiment, the wheat plants were collected from the field vegetation pots and brought to the Plant Physiology Laboratory from USAMV Cluj-Napoca to be assessed for another series of parameters. The morphological parameters of the plants, the lengths of the stems, spikes, and awns, were evaluated using the caliper method. The green and dry biomass of the stems and spikes was assessed using gravimetric methods in grams (g). For this purpose, they were oven-dried at 105 °C for 24 h. Samples were weighed several times and removed when similar consecutive weights were obtained. Subsequently, the total moisture content (MC) of the aerial parts of the wheat plants was evaluated using the formula

$$MC=\left(\frac{\left(Fresh\;biomass-Dry\;biomass\right)}{Fresh\;biomass}\right)\times 100$$

### 4.3. Data Analysis

The statistical data analysis was performed in RStudio v4.0.5 [59]. For basic statistics, the “psych” package was used [60], and the tables contained averages and standard errors along with Fisher LSD test different letters, which represent statistical differences at *p* < 0.05. The Fisher LSD test along with two-way and three-way ANOVA were done with the packages “agricolae” [61] and “broom” [62]. The interaction correlation charts were done with the “corrplot” package [63] after correlation detection by the “Hmisc” package [64]. A heatmap was done for a graphical abstract using the “pheatmap” package [65], and the cluster charts were done with the “ape” package [66]. The boxplot figures were extracted using the “ggplot2” package [67]. The boxplots contain the mean, median, standard deviation of the mean, and minimum and maximum values from a dataset. The above boxplots containing different letters from the LSD test are present to indicate the significant differences at *p* < 0.05.

To create the Discussion section, four types of large datasets were observed in interaction. Two present the physiological reaction, namely all of the stomata parameters and relative chlorophyll content regarding salinity, and two followed the visual aspects, namely morphological parameters seen in growth and development parameters and different biomasses assessed due to the applied saline doses.

The cluster analysis was used to highlight the similarities in the four complex datasets, following each variety based on the effect of salinity and ranking them within clusters. By analyzing one variety at a time in a cluster, the pattern of control and salinity doses tested positions the ecophysiological relevance of variation observed within the data and scored according to the distance observed in a singular specific cluster.

The salinity influence on the four sets of parameters that capture stomata characteristics, chlorophyll, growth and development elements, and biomass for each variety was framed in concentric circles, from the inside to the outside. Each circle was divided according to similar values into three segments, with maximum marked in green, medium associated with purple, and a blue segment representing the minimum. For each of the four circles, the control value was marked with a dot, and the different doses in the same circle segment symbolize its origin from the specific singular cluster.

## 5. Conclusions

This study highlights that the tolerance of varieties is not the result of a single parameter but of a coordinated reaction. The sunburst model permits the visualization of the most important findings of this study. The hierarchy based on four parameter classes identified Granny as the most stable variety, where the maintenance of the physiological parameters relative chlorophyll content and stomatal features directly correlated with higher biomass accumulation under salinity stress.

The Taisa wheat variety demonstrated high physiological plasticity, presenting a differentiated growth and development depending on the dose, which nevertheless allowed the maintenance of a higher biomass.

A uniform growth and development with increased stomatal characteristics but with decreased chlorophyll content resulted in variable biomass for Boema, ranking third place for this variety.

The only variety with overall decreased morphological parameters belonging to growth and development was Glosa. The other three classes of parameters were variably and irregularly influenced by different salinity doses.

The results of the study highlight the dose of 45 mM NaCl as a physiologically critical point for all tested varieties; at this level the largest stomatal opening was identified.

The use of the hierarchical model, visualized through sunburst diagrams, has proven to be a robust methodology for rapid germplasm screening. This approach allowed for the synthesis of multi-level data into a single image for each variety, facilitating the precise identification of resilient varieties by simultaneously integrating growth indicators and carbon allocation with stomatal dynamics and chlorophyll.

## Figures and Tables

**Table 1:** Stomata length (StoL) in μm of all wheat varieties depending on the saline solution concentrations: S1—control, S2—15 mM NaCl, S3—30 mM NaCl, S4—45 mM NaCl, S5—60 mM NaCl, S6—75 mM NaCl.

	Boema	Glosa	Granny	Taisa
S1	40.40 ± 0.73 d–i	40.13 ± 0.83 d–i	43.20 ± 0.82 abc	44.03 ± 0.52 a
S2	38.93 ± 0.59 hij	39.90 ± 0.68 e–i	37.03 ± 0.76 jk	42.13 ± 0.54 a–d
S3	39.60 ± 0.78 f–i	38.90 ± 0.80 hij	39.40 ± 0.57 f–i	41.20 ± 0.53 c–g
S4	40.23 ± 0.55 d–i	33.17 ± 0.70 l	38.33 ± 0.65 ijk	41.77 ± 0.58 b–e
S5	40.57 ± 0.66 d–h	41.80 ± 0.62 b–e	41.43 ± 0.68 b–f	43.47 ± 0.72 ab
S6	39.13 ± 0.60 g–j	41.00 ± 0.75 d–h	36.53 ± 0.64 k	41.90 ± 0.66 b–e

Note: Two-way ANOVA between V—different wheat varieties tested (F = 32.44, *p* < 0.001), S—different saline solutions (F = 3.43, *p* = 0.065), and V:S—interaction (F = 2.07, *p* = 0.150). Results are expressed as means ± SE, followed by distinct lowercase letters denoting statistical significance at *p* < 0.05 based on LSD test.

**Table 2:** Stomata width (StoW) in μm of all wheat varieties depending on the saline solution concentrations: S1—control, S2—15 mM NaCl, S3—30 mM NaCl, S4—45 mM NaCl, S5—60 mM NaCl, and S6—75 mM NaCl.

	Boema	Glosa	Granny	Taisa
S1	21.33 ± 0.41 e–i	19.80 ± 0.41 j	22.93 ± 0.40 bcd	22.97 ± 0.39 bc
S2	21.37 ± 0.44 e–h	19.80 ± 0.38 j	21.17 ± 0.46 e–i	21.57 ± 0.42 efg
S3	22.27 ± 0.61 b–e	20.63 ± 0.37 g–j	25.13 ± 0.57 a	21.87 ± 0.60 c–g
S4	22.07 ± 0.35 b–f	20.20 ± 0.42 hij	20.00 ± 0.31 ij	23.27 ± 0.41 b
S5	19.60 ± 0.48 jk	18.27 ± 0.48 k	22.20 ± 0.34 b–e	24.70 ± 0.35 a
S6	20.77 ± 0.31 f–j	20.77 ± 0.33 f–j	21.60 ± 0.34 d–g	21.80 ± 0.48 c–g

Note: Two-way ANOVA between V—different wheat varieties tested (F = 55.72, *p* < 0.001), S—different saline solutions (F = 2.26, *p* = 0.133), and V:S—interaction (F = 2.96, *p* = 0.082). Results are expressed as means ± SE, followed by distinct lowercase letters denoting statistical significance at *p* < 0.05 based on LSD test.

**Table 3:** Stomata pore aperture (StoAp) in μm of all wheat varieties depending on the saline solutions concentrations: S1—control, S2—15 mM NaCl, S3—30 mM NaCl, S4—45 mM NaCl, S5—60 mM NaCl, and S6—75 mM NaCl.

	Boema	Glosa	Granny	Taisa
S1	2.33 ± 0.18 b	1.93 ± 0.12 cde	2.10 ± 0.11 bcd	2.07 ± 0.14 bcd
S2	2.20 ± 0.09 bcd	1.93 ± 0.14 cde	1.87 ± 0.10 def	1.97 ± 0.09 b–e
S3	1.60 ± 0.11 efg	1.40 ± 0.09 g	2.23 ± 0.10 bcd	1.60 ± 0.10 efg
S4	2.73 ± 0.17 a	1.60 ± 0.11 efg	2.33 ± 0.10 b	1.40 ± 0.090 g
S5	1.67 ± 0.11 efg	1.50 ± 0.09 fg	2.17 ± 0.13 bcd	1.50 ± 0.09 fg
S6	2.33 ± 0.14 b	1.33 ± 0.09 g	2.30 ± 0.11 bc	2.17 ± 0.11 bcd

Note: Two-way ANOVA between V—different wheat varieties tested (F = 4.88, *p* = 0.028), S—different saline solutions (F = 2.74, *p* = 0.098), and V:S—interaction (F = 0.72, *p* = 0.396). Results are expressed as means ± SE, followed by distinct lowercase letters denoting statistical significance at *p* < 0.05 based on LSD test.

**Table 4:** Three-way ANOVA for stomata length (StoL), width (StoW) and aperture (StoAP) depending on the concentrations of the experimental independent variables wheat variety (V), saline solutions applied (S) and leaf sampling area (A).

	StoL		StoW		StoAP	
	*F*	*p Val*	*F*	*p Val*	*F*	*p Val*
**V**	33.11	<0.001 ***	57.11	<0.001 ***	4.88	0.028 *
**S**	3.50	0.062	2.32	0.128	2.74	0.098
**A**	7.51	0.006 **	14.23	<0.001 ***	0.89	0.345
**V:S**	2.12	0.146	3.04	0.082	0.72	0.396
**V:A**	3.69	0.055	0.70	0.404	1.09	0.298
**S:A**	1.26	0.262	6.89	0.009 **	0.57	0.450
**V:S:A**	6.38	0.012 *	0.04	0.849	1.55	0.213

Note: *F*—F statistics value, *p val*—*p* value, *—*p* < 0.05, **—*p* < 0.01 and ***—*p* < 0.001.

**Table 5:** Wheat leaf relative chlorophyll content (C1–C6) in SPAD units of all wheat varieties depending on the saline solution concentrations: S1—control, S2—15 mM NaCl, S3—30 mM NaCl, S4—45 mM NaCl, S5—60 mM NaCl, and S6—75 mM NaCl.

		Boema	Glosa	Granny	Taisa
C1	S1	18.33 ± 3.84 gh	49.83 ± 7.92 a–e	23.80 ± 2.80 fgh	19.27 ± 1.94 gh
	S2	15.33 ± 1.60 h	43.57 ± 3.85 b–f	54.07 ± 4.45 a–d	45.93 ± 1.83 b–f
	S3	36.70 ± 1.04 b–h	32.43 ± 5.00 d–h	28.17 ± 1.23 e–h	40.00 ± 4.04 b–g
	S4	35.47 ± 5.00 c–h	33.23 ± 5.04 c–h	25.63 ± 4.24 fgh	59.57 ± 4.52 ab
	S5	57.13 ± 5.37 abc	40.40 ± 0.23 b–g	24.67 ± 0.61 fgh	37.33 ± 3.84 b–h
	S6	26.27 ± 3.41 e–h	18.33 ± 2.11 gh	28.13 ± 1.62 e–h	71.03 ± 2.50 a
C2	S1	8.53 ± 1.23 d	16.37 ± 1.16 d	21.37 ± 1.62 cd	18.93 ± 0.63 d
	S2	10.83 ± 0.17 d	14.63 ± 2.11 d	23.23 ± 2.47 cd	29.37 ± 2.11 bcd
	S3	17.13 ± 1.62 d	59.40 ± 2.33 a	19.70 ± 1.55 cd	41.07 ± 2.00 abc
	S4	22.60 ± 0.60 cd	17.10 ± 1.20 d	24.40 ± 1.20 bcd	45.00 ± 7.99 ab
	S5	22.40 ± 0.72 cd	17.70 ± 2.67 d	15.87 ± 2.43 d	28.80 ± 3.76 bcd
	S6	13.47 ± 1.23 d	56.37 ± 3.24 a	14.70 ± 0.00 d	13.43 ± 2.43 d
C3	S1	52.27 ± 5.26 a–d	28.70 ± 0.60 fg	20.20 ± 4.63 g	31.77 ± 2.20 efg
	S2	28.77 ± 6.18 fg	32.40 ± 0.60 efg	46.33 ± 1.77 a–e	47.37 ± 3.27 a–e
	S3	46.50 ± 6.83 a–e	41.03 ± 3.07 b–f	43.37 ± 4.30 a–f	37.40 ± 0.70 d–g
	S4	53.87 ± 6.74 a–d	55.53 ± 5.89 abc	53.23 ± 3.20 a–d	44.70 ± 2.67 a–f
	S5	43.43 ± 6.21 a–f	52.43 ± 6.74 a–d	52.00 ± 2.22 a–d	57.53 ± 5.69 ab
	S6	37.30 ± 5.43 d–g	59.37 ± 3.73 a	38.53 ± 2.82 c–f	49.97 ± 4.59 a–d
C4	S1	44.77 ± 2.23 fgh	170.60 ± 0.30 a	99.07 ± 8.18 b–g	59.80 ± 3.90 d–h
	S2	108.57 ± 2.07 a–f	150.60 ± 1.25 ab	96.50 ± 3.08 b–h	51.60 ± 5.66 e–h
	S3	57.03 ± 7.42 d–h	127.40 ± 10.18 abc	115.13 ± 15.98 a–e	50.07 ± 4.46 e–h
	S4	67.33 ± 5.58 c–h	49.87 ± 4.98 e–h	80.57 ± 11.14 c–h	34.27 ± 6.49 gh
	S5	66.13 ± 0.03 c–h	73.23 ± 4.88 c–h	121.30 ± 19.47 a–d	32.67 ± 7.52 h
	S6	67.07 ± 6.67 c–h	108.40 ± 4.52 a–f	151.93 ± 12.56 ab	31.67 ± 8.15 h
C5	S1	69.77 ± 3.09 bc	41.97 ± 4.03 bcd	56.30 ± 2.55 bcd	41.23 ± 6.90 bcd
	S2	59.80 ± 7.95 bcd	34.63 ± 7.81 cd	35.47 ± 1.23 cd	36.60 ± 2.14 bcd
	S3	36.43 ± 4.36 bcd	32.43 ± 5.45 cd	48.37 ± 4.29 bcd	50.73 ± 1.59 bcd
	S4	25.07 ± 4.77 cd	32.23 ± 3.93 cd	44.20 ± 8.52 bcd	91.57 ± 10.77 b
	S5	37.30 ± 2.22 bcd	41.17 ± 4.45 bcd	22.00 ± 2.14 cd	194.20 ± 12.96 a
	S6	27.50 ± 2.80 cd	12.97 ± 2.97 d	17.10 ± 3.23 cd	14.67 ± 4.24 cd
C6	S1	11.57 ± 4.27 ef	33.00 ± 11.00 b–f	33.00 ± 7.65 b–f	11.60 ± 2.67 ef
	S2	15.87 ± 3.24 def	11.00 ± 2.14 ef	36.70 ± 0.00 a–e	31.20 ± 7.45 c–f
	S3	7.30 ± 2.14 ef	42.20 ± 5.50 a–d	28.13 ± 4.41 c–f	11.00 ± 2.14 ef
	S4	8.53 ± 2.20 ef	7.90 ± 2.67 ef	50.80 ± 4.03 abc	52.63 ± 12.82 abc
	S5	18.10 ± 9.01 def	28.77 ± 4.26 c–f	61.17 ± 9.29 ab	42.87 ± 10.56 a–d
	S6	6.57 ± 2.26 f	63.67 ± 7.90 a	27.23 ± 7.16 c–f	20.43 ± 2.65 def

Note: Results are expressed as means ± SE, followed by distinct lowercase letters denoting statistical significance at *p* < 0.05 based on LSD test.

**Table 6:** Plant height (PlantH) in cm of all wheat varieties depending on the saline solution concentrations: S1—control, S2—15 mM NaCl, S3—30 mM NaCl, S4—45 mM NaCl, S5—60 mM NaCl, and S6—75 mM NaCl.

	Boema	Glosa	Granny	Taisa
S1	39.43 ± 0.30 b–e	49.87 ± 1.88 a	45.20 ± 2.20 ab	29.17 ± 1.94 f
S2	40.67 ± 0.81 b–e	41.73 ± 0.91 b–e	44.47 ± 2.09 a–d	42.27 ± 2.14 b–e
S3	43.57 ± 2.60 a–d	41.33 ± 1.19 b–e	45.83 ± 0.85 ab	44.73 ± 2.55 abc
S4	37.67 ± 0.68 cde	42.20 ± 3.18 b–e	45.93 ± 1.01 ab	45.20 ± 1.04 ab
S5	35.27 ± 1.05 ef	39.33 ± 1.20 b–e	45.77 ± 1.83 ab	44.00 ± 1.89 a–d
S6	39.97 ± 2.02 b–e	37.47 ± 1.03 de	43.37 ± 1.48 a–d	43.83 ± 1.99 a–d

Note: Two-way ANOVA between V—different wheat varieties tested (F = 4.07, *p* = 0.048), S—different saline solutions (F = 0.11, *p* = 0.746) and V:S—interaction (F = 14.01, *p* < 0.001). Results are expressed as means ± SE, followed by distinct lowercase letters denoting statistical significance at *p* < 0.05 based on LSD test.

**Table 7:** Spike length (S_L) and awn length (A_L) in cm of all wheat varieties depending on the saline solution concentrations: S1—control, S2—15 mM NaCl, S3—30 mM NaCl, S4—45 mM NaCl, S5—60 mM NaCl, and S6—75 mM NaCl.

	Boema	Glosa	Granny	Taisa
Spike				
S1	8.27 ± 0.44 a–d	7.50 ± 0.32 a–h	7.10 ± 0.26 c–i	7.00 ± 0.30 c–i
S2	8.10 ± 0.61 a–e	6.87 ± 0.33 e–i	6.93 ± 0.27 d–i	6.37 ± 0.17 hi
S3	6.03 ± 0.47 i	6.83 ± 0.32 e–i	8.03 ± 0.09 a–e	7.93 ± 0.27 a–f
S4	7.87 ± 0.72 a–g	6.93 ± 0.35 d–i	7.27 ± 0.07 b–i	8.27 ± 0.12 a–d
S5	7.40 ± 0.44 b–i	6.53 ± 0.23 ghi	7.33 ± 0.33 b–i	8.80 ± 0.61 a
S6	8.53 ± 0.30 ab	6.60 ± 0.20 f–i	8.37 ± 0.30 abc	7.93 ± 0.23 a–f
Two-way ANOVA: V (F = 0.56, *p* = 0.457); S (F = 3.15, *p* = 0.080); V:S (F = 6.10, *p* = 0.016)				
Awns				
S1	5.03 ± 0.12 a	3.53 ± 0.22 d–g	3.40 ± 0.29 efg	4.23 ± 0.26 a–f
S2	4.93 ± 0.48 ab	3.33 ± 0.15 efg	2.80 ± 0.15 g	2.47 ± 0.09 g
S3	3.77 ± 0.28 a–g	3.67 ± 0.28 b–g	3.73 ± 0.27 a–g	4.27 ± 0.24 a–f
S4	4.87 ± 0.28 abc	3.60 ± 0.46 c–g	3.07 ± 0.09 fg	4.33 ± 0.19 a–f
S5	4.73 ± 0.52 a–d	3.20 ± 0.10 efg	2.60 ± 0.12 g	4.27 ± 0.29 a–f
S6	4.87 ± 0.03 abc	3.13 ± 0.18 efg	3.60 ± 0.31 c–g	4.40 ± 0.06 a–e
Two-way ANOVA: V (F = 7.58, *p* = 0.008); S (F = 0.17, *p* = 0.678); V:S (F = 1.63, *p* = 0.206)				

Note: Two-way ANOVA between V—different wheat varieties tested, S—different saline solutions and V:S—interaction. Results are expressed as means ± SE, followed by distinct lowercase letters denoting statistical significance at *p* < 0.05 based on LSD test.

**Table 8:** Spike (Sp_no) number of all wheat varieties depending on the saline solution concentrations: S1—control, S2—15 mM NaCl, S3—30 mM NaCl, S4—45 mM NaCl, S5—60 mM NaCl, and S6—75 mM NaCl.

	Boema	Glosa	Granny	Taisa
S1	20.67 ± 0.88 abc	19.67 ± 0.88 abc	24.33 ± 0.88 a	2.33 ± 0.88 d
S2	25.67 ± 0.88 a	21.67 ± 0.88 ab	25.00 ± 1.15 a	18.33 ± 1.45 abc
S3	19.33 ± 0.33 abc	22.67 ± 0.88 ab	26.67 ± 0.88 a	21.67 ± 0.33 ab
S4	15.67 ± 0.88 bc	25.67 ± 0.88 a	21.67 ± 1.45 ab	14.67 ± 0.88 bc
S5	21.00 ± 1.15 abc	25.67 ± 1.45 a	24.33 ± 1.45 a	12.67 ± 1.45 c
S6	22.67 ± 1.45 ab	22.33 ± 1.20 ab	26.33 ± 1.45 a	21.67 ± 1.45 ab

Note: Two-way ANOVA between V—different wheat varieties tested (F = 7.75, *p* = 0.007), S—different saline solutions (F = 3.78, *p* = 0.056) and V:S—interaction (F = 3.79, *p* = 0.056). Results are expressed as means ± SE, followed by distinct lowercase letters denoting statistical significance at *p* < 0.05 based on LSD test.

**Table 9:** Heading percentages (Spp) of all wheat varieties depending on the saline solution concentrations: S1—control, S2—15 mM NaCl, S3—30 mM NaCl, S4—45 mM NaCl, S5—60 mM NaCl, and S6—75 mM NaCl.

	Boema	Glosa	Granny	Taisa
S1	70.92 ± 5.85 bc	77.74 ± 4.41 abc	89 ± 0.35 abc	11.53 ± 3.97 d
S2	89.61 ± 3.95 abc	87.78 ± 2.50 abc	91.58 ± 5.18 ab	71.23 ± 3.36 bc
S3	79.57 ± 1.65 abc	90.87 ± 5.05 ab	94.12 ± 1.17 ab	85.09 ± 5.97 abc
S4	64.37 ± 7.34 c	96.24 ± 0.13 ab	87.72 ± 2.68 abc	79.10 ± 3.24 abc
S5	74.03 ± 2.70 abc	97.33 ± 2.67 a	95.89 ± 2.41 ab	85.78 ± 4.88 abc
S6	79.86 ± 3.51 abc	86.89 ± 1.78 abc	91.74 ± 2.47 ab	86.12 ± 8.60 abc

Note: Two-way ANOVA between V—different wheat varieties tested (F = 1.11, *p* = 0.296), S—different saline solutions (F = 10.42, *p* = 0.002) and V:S—interaction (F = 12.82, *p* < 0.001). Results are expressed as means ± SE, followed by distinct lowercase letters denoting statistical significance at *p* < 0.05 based on LSD test.

## Data Availability

The original contributions presented in this study are included in this article. Further inquiries can be directed to the main and the corresponding author.

## Abbreviations

The following abbreviations are used in this manuscript:

V	Wheat variety
S	Tested saline solution
Bo	Boema wheat variety
Gl	Glosa wheat variety
Gr	Granny wheat variety
S1	Control—without salinity stress
S2	Lowest salinity solution with a concentration of 15 mM NaCl
S3	Low salinity solution with a concentration of 30 mM NaCl
S4	Medium salinity solution with a concentration of 45 mM NaCl
S5	Increased salinity solution with a concentration of 60 mM NaCl
S6	Highest salinity solution with a concentration of 75 mM NaCl
SD	Stomata density
N	Number of stomata
A	Leaf sampling area
A1	Base of the leaf
A2	Middle of the leaf
A3	Tip of the leaf
StoL	Stomata length
StoW	Stomata width
StoAp	Stomata pore aperture
SD_A1	Stomata density for the leaf base
SD_A2	Stomata density for the middle of the leaf
SD_A3	Stomata density for the leaf tip
StoL_A1	Stomata length assessed in the leaf base
StoL_A2	Stomata length assessed in the middle of the leaf
StoL_A3	Stomata length assessed in the tip leaf
StoW_A1	Stomata width assessed in the leaf base
StoW_A1	Stomata width assessed in the middle of the leaf
StoW_A1	Stomata width assessed in the tip leaf
StoAp_A1	Stomata pore aperture assessed in the leaf base
StoAp_A2	Stomata pore aperture assessed in the middle of the leaf
StoAp_A3	Stomata pore aperture assessed in the tip leaf
C1	First assessment of leaf relative chlorophyll content
C2	Second assessment of leaf relative chlorophyll content
C3	Third assessment of leaf relative chlorophyll content
C4	Fourth assessment of leaf relative chlorophyll content
C5	Fifth assessment of leaf relative chlorophyll content
C6	Sixth assessment of leaf relative chlorophyll content
C_Sp	Spike relative chlorophyll content
PlantH	Plant height
S_L	Spike length
A_L	Awn length
Sp_no	Number of spikes
ShFB	Shoot fresh biomass
SpFB	Spike fresh biomass
ShDB	Shoot dry biomass
SpDB	Spike dry biomass
ShMC	Shoot moisture content
SpMC	Spike moisture content
Spp	Heading percentage

## References

[1] Ilbery B.W., Bowler I.R. (2003). Industrialization and World Agriculture. Companion Encyclopedia of Geography.

[2] Kremen C., Iles A., Bacon C. (2012). Diversified Farming Systems: An Agroecological, Systems-Based Alternative to Modern Industrial Agriculture. Ecol. Soc..

[3] Welch R.M., Graham R.D. (1999). A New Paradigm for World Agriculture: Meeting Human Needs: Productive, Sustainable, Nutritious. Field Crops Res..

[4] Altieri M.A., Nicholls C.I. (2017). The Adaptation and Mitigation Potential of Traditional Agriculture in a Changing Climate. Clim. Chang..

[5] Mukhopadhyay R., Sarkar B., Jat H.S., Sharma P.C., Bolan N.S. (2021). Soil Salinity under Climate Change: Challenges for Sustainable Agriculture and Food Security. J. Environ. Manag..

[6] Cheeseman J. (2016). Food Security in the Face of Salinity, Drought, Climate Change, and Population Growth. Halophytes for Food Security in Dry Lands.

[7] Dwivedi S.L., Scheben A., Edwards D., Spillane C., Ortiz R. (2017). Assessing and Exploiting Functional Diversity in Germplasm Pools to Enhance Abiotic Stress Adaptation and Yield in Cereals and Food Legumes. Front. Plant Sci..

[8] Marone D., Russo M.A., Mores A., Ficco D.B., Laidò G., Mastrangelo A.M., Borrelli G.M. (2021). Importance of Landraces in Cereal Breeding for Stress Tolerance. Plants.

[9] Ghosh A., Kumar A., Biswas G. (2024). Exponential Population Growth and Global Food Security: Challenges and Alternatives. Bioremediation of Emerging Contaminants from Soils.

[10] Basso B., Liu L., Ritchie J.T. (2016). A Comprehensive Review of the CERES-Wheat,-Maize and-Rice Models’ Performances. Adv. Agron..

[11] Hafner S. (2003). Trends in Maize, Rice, and Wheat Yields for 188 Nations over the Past 40 Years: A Prevalence of Linear Growth. Agric. Ecosyst. Environ..

[12] Lintas C., Mariani-Costantini A. (1991). Cereal Foods: Wheat, Corn, Rice, Barley, and Other Cereals and Their Products. The Mediterranean Diets in Health and Disease.

[13] Grote U., Fasse A., Nguyen T.T., Erenstein O. (2021). Food Security and the Dynamics of Wheat and Maize Value Chains in Africa and Asia. Front. Sustain. Food Syst..

[14] Wassmann R., Jagadish S.V.K., Sumfleth K., Pathak H., Howell G., Ismail A., Heuer S. (2009). Regional Vulnerability of Climate Change Impacts on Asian Rice Production and Scope for Adaptation. Adv. Agron..

[15] Mendes K.R., Bonifacio A., Martins M.O., Sousa R.H., Monteiro M.V., Silveira J.A. (2024). Heat Shock Combined with Salinity Impairs Photosynthesis but Stimulates Antioxidant Defense in Rice Plants. Environ. Exp. Bot..

[16] Burlakoti S., Devkota A.R., Poudyal S., Kaundal A. (2024). Beneficial Plant–Microbe Interactions and Stress Tolerance in Maize. Appl. Microbiol..

[17] Nauman M., Alghamdi A.A., Ahmed J., Ahmad R., Bilal M., Khan S.A., Shahzad M. (2025). Enhanced Resilience to Salt Stress: An Integrated Approach Addressing Physiochemical Attributes of Wheat (*Triticum aestivum* L.). Pol. J. Environ. Stud..

[18] Naeem M., Iqbal M., Shakeel A., Ul-Allah S., Hussain M., Rehman A., Ashraf M. (2020). Genetic Basis of Ion Exclusion in Salinity Stressed Wheat: Implications in Improving Crop Yield. Plant Growth Regul..

[19] Saddiq M.S., Iqbal S., Hafeez M.B., Ibrahim A.M., Raza A., Fatima E.M., Ciarmiello L.F. (2021). Effect of Salinity Stress on Physiological Changes in Winter and Spring Wheat. Agronomy.

[20] Gul Z., Tang Z.H., Arif M., Ye Z. (2022). An Insight into Abiotic Stress and Influx Tolerance Mechanisms in Plants to Cope in Saline Environments. Biology.

[21] Velicevici G., Madosa E., Oproi E., Iordanescu O., Dragomir P. (2023). The Effect of Salinity on the Chlorophyll Content of Wheat. J. Cent. Eur. Green Innov..

[22] Mbarki S., Sytar O., Cerda A., Zivcak M., Rastogi A., He X., Brestic M. (2018). Strategies to Mitigate the Salt Stress Effects on Photosynthetic Apparatus and Productivity of Crop Plants. Salin. Responses Toler. Plants.

[23] Pan T., Liu M., Kreslavski V.D., Zharmukhamedov S.K., Nie C., Yu M., Shabala S. (2021). Non-Stomatal Limitation of Photosynthesis by Soil Salinity. Crit. Rev. Environ. Sci. Technol..

[24] Azizpour K., Shakiba M.R., Sima N.K.K., Alyari H., Mogaddam M., Esfandiari E., Pessarakli M. (2010). Physiological Response of Spring Durum Wheat Genotypes to Salinity. J. Plant Nutr..

[25] Shokri N., Hassani A., Sahimi M. (2024). Multi-scale Soil Salinization Dynamics from Global to Pore Scale: A Review. Rev. Geophys..

[26] Desheva G., Sabeva M., Zacharieva M. (2016). Variation of Agronomic Traits among Introduced Winter Bread Wheat Cultivars. Trakia J. Sci..

[27] Hussain S., Shaukat M., Ashraf M., Zhu C., Jin Q., Zhang J. (2019). Salinity Stress in Arid and Semi-Arid Climates: Effects and Management in Field Crops. Climate Change and Agriculture.

[28] Wang Z., Poudyal S., Kopp K., Zhang Y. (2025). Response of Ornamental Plants to Salinity: Impact on Species-Specific Growth, Visual Quality, Photosynthetic Parameters, and Ion Uptake. Front. Plant Sci..

[29] Mane A.V., Deshpande T.V., Wagh V.B., Karadge B., Samant J.S. (2011). A Critical Review on Physiological Changes Associated with Reference to Salinity. Int. J. Environ. Sci..

[30] Seleiman M.F., Aslam M.T., Alhammad B.A., Hassan M.U., Maqbool R., Chattha M.U., Battaglia M.L. (2022). Salinity stress in wheat: Effects, mechanisms and management strategies. Phyton-Int. J. Exp. Bot..

[31] Tao R., Ding J., Li C., Zhu X., Guo W., Zhu M. (2021). Evaluating and Screening of Agro-Physiological Indices for Salinity Stress Tolerance in Wheat at the Seedling Stage. Front. Plant Sci..

[32] Hasanuzzaman M., Zhou M., Shabala S. (2023). How Does Stomatal Density and Residual Transpiration Contribute to Osmotic Stress Tolerance?. Plants.

[33] Guo R., Shi L., Yang Y. (2009). Germination, Growth, Osmotic Adjustment and Ionic Balance of Wheat in Response to Saline and Alkaline Stresses. Soil Sci. Plant Nutr..

[34] Turan S., Tripathy B.C. (2015). Salt-stress induced modulation of chlorophyll biosynthesis during de-etiolation of rice seedlings. Physiol. Plant..

[35] Trușcă M., Stoian V., Vâtcă A., Stoian V., Vidican R., Păcurar F., Vâtcă S. (2025). Critical Salinity Thresholds Impacting Wheat Germination: Determining Dose-Dependent Responses Across Varieties. Sci. Papers. Ser. A Agron..

[36] Trușcă M., Gâdea Ș., Vâtcă A., Stoian V., Racz I., Vâtcă S. (2024). Morpho-Physiological Characteristics under Salinity Stress of *Triticum aestivum* L. Var. Alex. Bull. Univ. Agric. Sci. Vet. Med. Cluj-Napoca. Agric..

[37] Karimi S.M., Freund M., Wager B.M., Knoblauch M., Fromm J., Mueller H.M., Ache P., Krischke M., Mueller M.J., Müller T. (2021). Under Salt Stress Guard Cells Rewire Ion Transport and Abscisic Acid Signaling. New Phytol..

[38] Caine R.S., Harrison E.L., Sloan J., Flis P.M., Fischer S., Khan M.S., Croft H. (2023). The Influences of Stomatal Size and Density on Rice Abiotic Stress Resilience. New Phytol..

[39] Marinciu C.M., Monica T.A.N.C., Şerban G., Mandea V., Toncea I., Petcu V., Săulescu N. (2022). Performance of Some Romanian Winter Wheat Cultivars under Organic Agriculture Conditions I. Grain yield. Ann. Univ. Craiova-Agric. Mont. Cadastre Ser..

[40] Brăilă M., Trifan D., Ghiorghe A.I., Mihalache M. (2025). Studies on the Adaptability of Some Romanian Varieties of Autumn Wheat to the Current Climate Changes in Northern Bărăgan. Sci. Papers. Ser. A. Agron..

[41] Târșoagă L., Păunescu A., Horoiaș R., Ilie L. (2025). Studies on the Influence of Input Application on the Productivity of Romanian Wheat Varieties, at Ards Caracal. Sci. Papers. Ser. A Agron..

[42] Mureșan D., Varadi A., Racz I., Kadar R., Duda M.M. (2019). The Behavior of Several Facultative Wheat Genotypes Sown in Spring. Res. J. Agric. Sci..

[43] Chețan F., Hirișcău D., Rusu T., Bărdaș M., Chețan C., Șimon A., Moraru P.I. (2024). Yield, Protein Content and Water-Related Physiologies of Spring Wheat Affected by Fertilizer System and Weather Conditions. Agronomy.

[44] Sharma S., Tiwari G. (2022). A Practical Manual on Fundamentals of Plant Physiology.

[45] Negrão S.M.S., Tester M. (2017). Evaluating Physiological Responses of Plants to Salinity Stress. Ann. Bot..

[46] Lungu E., Turek-Rahoveanu M.M. (2023). Research on Agroecological Zoning for Winter Wheat (*Triticum aestivum* L.) in South-Eastern Romania. Sci. Pap. Ser. Manag. Econ. Eng. Agric. Rural. Dev..

[47] Mandici A., Cretu D.E., Burlica R., Astanei D., Beniuga O., Rosu C., Miron A. (2022). Preliminary Study on the Impact of Non-Thermal Plasma Activated Water on the Quality of *Triticum aestivum* L. Cv. Glosa sprouts. Horticulturae.

[48] Lindberg S., Premkumar A. (2023). Ion Changes and Signaling under Salt Stress in Wheat and Other Important Crops. Plants.

[49] Keisham M., Mukherjee S., Bhatla S.C. (2018). Mechanisms of Sodium Transport in Plants—Progresses and Challenges. Int. J. Mol. Sci..

[50] Ibrahimova U., Zivcak M., Gasparovic K., Rastogi A., Allakhverdiev S.I., Yang X., Brestic M. (2021). Electron and Proton Transport in Wheat Exposed to Salt Stress: Is the Increase of the Thylakoid Membrane Proton Conductivity Responsible for Decreasing the Photosynthetic Activity in Sensitive Genotypes?. Photosynth. Res..

[51] El-Hendawy S., Dewir Y.H., Elsayed S., Schmidhalter U., Al-Gaadi K., Tola E., Refay Y., Tahir M.U., Hassan W.M. (2022). Combining Hyperspectral Reflectance Indices and Multivariate Analysis to Estimate Different Units of Chlorophyll Content of Spring Wheat under Salinity Conditions. Plants.

[52] Racz I., Berindean I.V., Kadar R., Hiriṣcău D., Varadi A., Morar D., Andraș B. (2024). The Variability of Quantitative Traits Parameters of Facultative Wheat Affected by Sowing Time. Bull. Univ. Agric. Sci. Vet. Med. Cluj-Napoca. Agric..

[53] Iacob I.N., Roșculete C.A., Păunescu R.A., Bonciu E. (2025). Research on the Response to Prolonged Drought of an Assortment of Wheat Varieties, Through the Rate of Water Loss From the Flag Leaf, on the Chernozem of Caracal. Sci. Papers. Ser. A Agron..

[54] Trușcă M., Stoian V., Gâdea Ș., Vâtcă A., Jug I., Brozović B., Vâtcă S. (2024). Chlorophyll Content. Phenol. Morphol. Trait. Wheat Under Salin. Stress. Ser. A Agron..

[55] NIARD Fundulea. https://www.incda-fundulea.ro/fise/fise.html.

[56] Melucã C., Sturzu R., Cojocaru J.M. (2021). Behavior of Local and Foreign Winter Wheat Varieties at Different Sowing Densities in the Southern Part of the Country. Life Sci. Sustain. Dev..

[57] Konvalina P., Capouchová I., Stehno Z., Moudry J. (2010). Agronomic Characteristics of the Spring Forms of the Wheat Landraces (Einkorn, Emmer, Spelt, Intermediate Bread Wheat) Grown in Organic Farming. J. Agrobiol..

[58] Bobková L., Granny S.W. (2004). New Varieties NovÈ odrdy. Czech J. Genet. Plant Breed.

[59] R Core Team (2024). R: A Language and Environment for Statistical Computing.

[60] Revelle W. (2024). R Package.

[61] De Mendiburu F. (2023). R Package.

[62] Robinson D., Hayes A., Couch S. (2024). R Package.

[63] Wei T., Simko V. (2024). R Package.

[64] Harrell F. (2025). R Package.

[65] Kolde R. (2025). R package.

[66] Paradis E., Schliep K. (2019). ape 5.0: An environment for modern phylogenetics and evolutionary analyses in R. Bioinformatics.

[67] Wickham H. (2016). Ggplot2: Elegant Graphics for Data Analysis.

